# Analysing the effect caused by increasing the molecular volume in M1-AChR receptor agonists and antagonists: a structural and computational study†

**DOI:** 10.1039/d3ra07380g

**Published:** 2024-03-14

**Authors:** Wilber Montejo-López, Raúl Sampieri-Cabrera, María Inés Nicolás-Vázquez, Juan Manuel Aceves-Hernández, Rodrigo Said Razo-Hernández

**Affiliations:** a Departamento de Ciencias Químicas, Facultad de Estudios Superiores Cuautitlán Campo 1, Universidad Nacional Autónoma de México Avenida 1o de Mayo s/n, Colonia Santa María las Torres Cuautitlán Izcalli Estado de Mexico 54740 Mexico wmontejo61@gmail.com nicovain@yahoo.com.mx; b Departamento de Fisiología, Facultad de Medicina, Universidad Nacional Autónoma de México, Centro de Ciencias de Complejidad, Universidad Nacional Autónoma de México Mexico sampieri@comunidad.unam.mx; c Unidad de Investigación Multidisciplinaria L14 (Alimentos, Micotoxinas, y Micotoxicosis), Facultad de Estudios Superiores Cuautitlán, Universidad Nacional Autónoma de México Cuautitlán Izcalli Estado de Mexico 54714 Mexico juanmanuel.is.acevesh@gmail.com; d Centro de Investigación en Dinámica Celular, Instituto de Investigación en Ciencias Básicas y Aplicadas, Universidad Autónoma del Estado de Morelos Av. Universidad 1001 Cuernavaca 62209 Mexico rodrigo.razo@uaem.mx

## Abstract

M1 muscarinic acetylcholine receptor (M1-AChR), a member of the G protein-coupled receptors (GPCR) family, plays a crucial role in learning and memory, making it an important drug target for Alzheimer's disease (AD) and schizophrenia. M1-AChR activation and deactivation have shown modifying effects in AD and PD preclinical models, respectively. However, understanding the pharmacology associated with M1-AChR activation or deactivation is complex, because of the low selectivity among muscarinic subtypes, hampering their therapeutic applications. In this regard, we constructed two quantitative structure–activity relationship (QSAR) models, one for M1-AChR agonists (total and partial), and the other for the antagonists. The binding mode of 59 structurally different compounds, including agonists and antagonists with experimental binding affinity values (pKi), were analyzed employing computational molecular docking over different structures of M1-AChR. Furthermore, we considered the interaction energy (*E*_inter_), the number of rotatable bonds (NRB), and lipophilicity (ilogP) for the construction of the QSAR model for agonists (*R*^2^ = 89.64, *Q*_LMO_^2^ = 78, and *Q*_ext_^2^ = 79.1). For the QSAR model of antagonists (*R*^2^ = 88.44, *Q*_LMO_^2^ = 82, and *Q*_ext_^2^ = 78.1) we considered the *E*_inter_, the fraction of sp^3^ carbons *f*Csp^3^, and lipophilicity (Mlog*P*). Our results suggest that the ligand volume is a determinant to establish its biological activity (agonist or antagonist), causing changes in binding energy, and determining the affinity for M1-AChR.

## Abstract 2

Two QSAR models which correlates the interaction energy and structural features of agonists and antagonists of M1-AChR.

## Graphical abstract

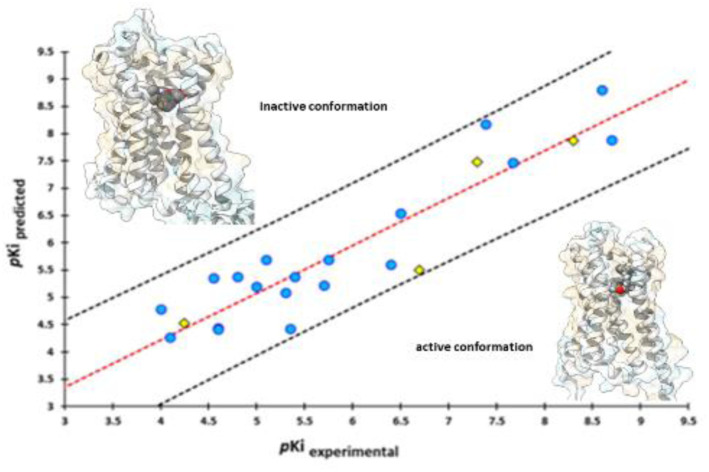

## Introduction

G protein-coupled receptors (GPCRs) are a functionally and topologically diverse superfamily of receptors and represent the largest protein family encoded by the human genome. GPCRs contain seven membrane-crossing α-helices and are highly conformationally dynamic, playing a pivotal role in intracellular communication, translating signals from extracellular ligands to intracellular effectors, to regulate numerous physiological processes. The protein property of seven transmembrane helices (7TM) endows them with easy access, while diversified downstream signalling pathways make them attractive for drug development.^1^ The human genome encodes more than 800 different GPCRs, revealing their importance in most physiological cellular processes, and making them viable targets for therapeutic intervention in numerous diseases.^2^ GPCRs are of great pharmaceutical relevance as their ligands account for 35% of currently marketed drugs.^3^

Muscarinic acetylcholine receptors (mAChR) are a subclass of the class A GPCR family, comprising 5 subtypes (M1–M5), which are encoded by distinct CHRM genes (CHRM1–CHRM5) and are involved in a variety of physiological functions.^4,5^ Among the five muscarinic receptor subtypes (M1R–M5R), M1R, M3R, and M5R are coupled to protein Gq/11, whereas M2R and M4R preferentially signal through protein alpha subunits, Gi/o.^6,7^ M1R and M4R are associated with learning, memory, and cognition and are promising targets for the treatment of neurological disorders.^8–10^ Abnormal muscarinic receptor function has been shown to correlate with Alzheimer's disease (AD), Parkinson's disease, schizophrenia, and epilepsy.^11^ Loss of cholinergic neurotransmission in the cerebellar cortex and other brain regions contributes to decreased cognitive function in AD.^12^

AD is a devastating progressive neurodegenerative disorder that slowly destroys memory and thinking skills; neurons in the hippocampus and entorhinal cortex are the first to degenerate (Kempuraj, 2016).^13^ Classic AD pathologies are distinguished primarily by the accumulation of amyloid-β peptide (Aβ) and neurofibrillary tangles (NFT),^14^ intraneuronal tangles of hyperphosphorylated microtubule-associated tau protein,^15^ and synaptic loss.^16,17^ Dysregulation of mAChR is a hallmark of progressive AD pathology.^18^ M1-AChR is widely regarded as a key receptor or mediator of cognitive function and thus a target for AD treatment.^19^ Currently, there are no treatments that can delay the progression of AD. However, there is evidence that M1-AChR activation can not only restore memory loss in AD patients but can also delay the progression of neurodegenerative disease in preclinical animal models.^20,21^

M1-AChR constitutes up to 60% of total mAChR expression in the central nervous system (CNS) and is abundantly expressed in major areas of the prosencephalon, including the hippocampus, neostriatum and cerebral cortex.^22,23^ The neuroprotective properties of cholinergic stimulation in the brain are mainly attributed to the activation of the M1 subtype.^24,25^ Genetic ablation or pharmacological inhibition of M1 muscarinic acetylcholine receptor (M1-AChR) signalling in rodents results in significant cognitive deficits.^26–28^ On the other hand, M1-AChR activation rescues learning and memory deficits in preclinical models of neurodegeneration and in human patients with CNS disorders such as schizophrenia.^26,29,30^ In a preclinical mouse model of AD, activation of M1-AChR with an orthosteric ligand can regulate the proteolytic processing of amyloid precursor protein, which reduces the appearance of Aβ plaques.^11^

Crystal structures resolution of the five mAChR subtypes^31–33^ have reaffirmed previous phylogenetic analyses of a highly structurally conserved hydrophilic cavity deep within regions 2–7 of the transmembrane (TM) unit of the five subtypes.^34^ Acetylcholine (ACh) binds to amino acid residues located in the outer region of this orthosteric binding pocket through an ionic interaction between the positively charged polar head group of ACh and the negatively charged aspartate residue within TM3.^35^ Importantly, the high sequence identities among these orthosteric pockets have been a significant limitation in designing subtype-selective ligands for these receptors. Therefore, new strategies are being pursued in AD therapy to selectively activate M1-AChR, through the development of highly selective agonists for M1R or the use of positive allosteric modulators, which selectively enhance the affinity of the M1 receptor for acetylcholine.^26,36^

Quantitative structure–activity relationship (QSAR) models are applied to determine the relationship between molecular properties and observed pharmacological activities of a group of congeneric compounds.^37,38^ QSAR relationship combined with molecular docking is a powerful tool for developing new drug candidates.^39–41^ Molecular docking reveals a ligand's predominant binding modes with the known receptor structure. Therefore, the method can identify the correct positions of ligands in the binding pocket of a protein and predict the affinity between ligand and protein.^42,43^

Recent insights into the physiology, pharmacology, and structure of muscarinic receptors in active and inactive conformations aid in the development of new selective ligands for muscarinic receptor subtypes that could prove useful as treatments for many serious pathophysiological conditions. Available X-ray structures together with cryo-electron microscopy (cryo-EM) structures of the human M1 muscarinic receptor M1 allow an investigation of drug–receptor interactions at the molecular level. Taking advantage of the available data on M1-AChR and its agonists/antagonists, we performed QSAR studies to study the effect of the molecular volume on the biological activity of these compounds. Using this information, we employed computational molecular docking to correlate the QSAR results with the binding of these compounds over M1-AChR. Finally, combining the docking results with other molecular descriptors related to the structure and solubility of the compounds we obtained two mathematical models for the prediction of the pKi of M1-AChR agonists or antagonists.

## Materials and methods

### Compounds preparation

**Fig. 1:**
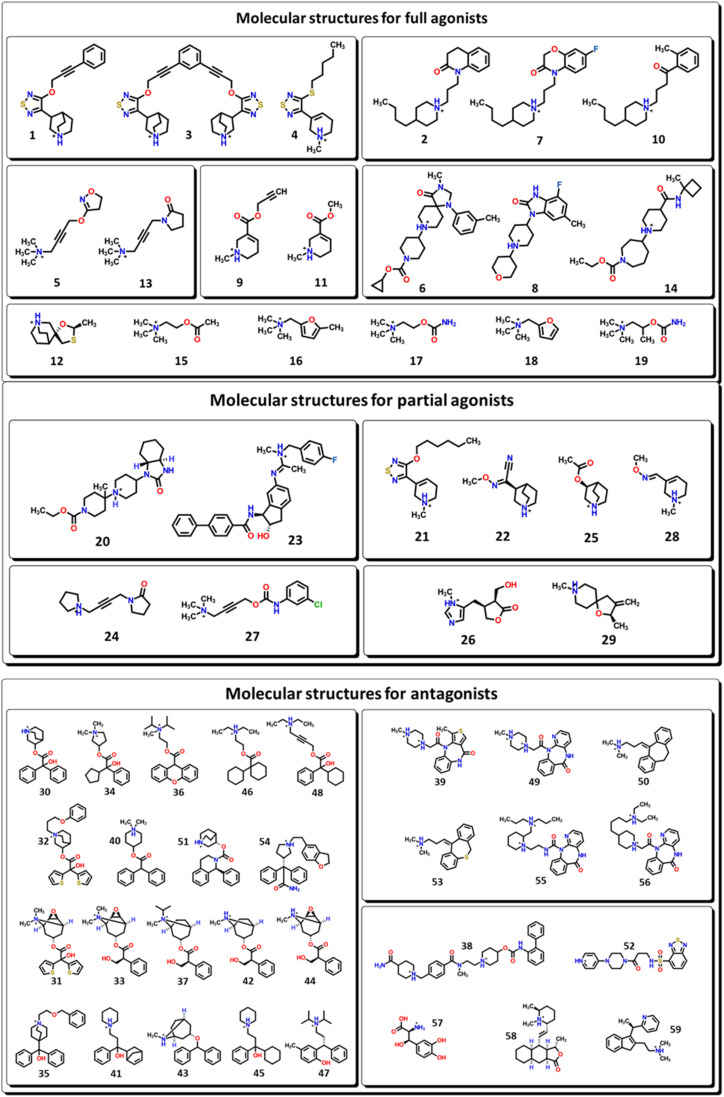
2D representations of all the biologically active molecules considered for this work.

The biological active compounds were selected from the CHEMBL database^44^ based on the availability of its protein–ligand binding affinity (pKi) value as a measure of its activity towards M1-AChR. In total, 59 biologicals active compounds were obtained: antagonists (30 compounds), full agonists (18 compounds), and partial agonists (11 compounds) (Fig. 1). The 2D structures of the compounds were drawn using ChemDraw ultra-version 15.0 software and then transformed to 3D format using Spartan v0.6 software. Chemical structures were minimized by molecular mechanics force field counting (MM+) to eliminate deformation energy and ensure a well-defined conformational relationship between the molecules used in this study. Furthermore, the optimization geometry of the molecules was performed in the Spartan interface by using density functional theory (DFT) with the B3LYP/631-G* basis set.^45^

### QSAR study

After analysing the chemical structure of all the biological compounds by categories, we identified that a correlation between the molecular volume and the biological activity (pKi) of these compounds was possible. Therefore, we used the molecular volume obtained from the optimized structures and generated a mathematical equation for the agonist and antagonist categories. To accomplish this, we used the single ordinary least-squares model regression analysis, using Excel Microsoft Office 365. To evaluate the confidence and the predictive ability of the QSAR models we used different statistical parameters *R*^2^, *Q*^2^, *s*, *F*, like in other works made by our group.^40,46^

### Computational molecular docking

**Fig. 2:**
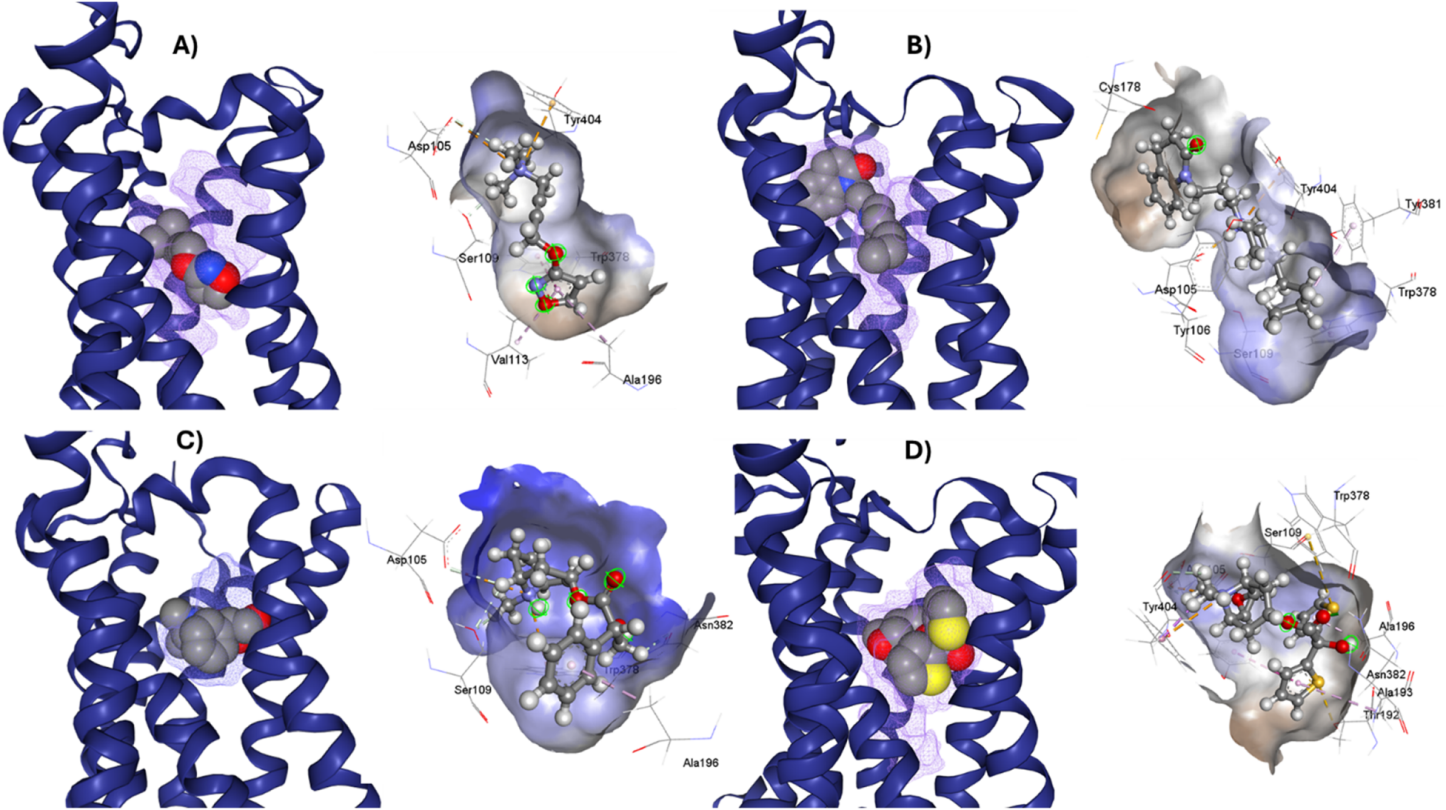
Ligands binding site for specific (sub) pockets at the muscarinic receptor. (A) cryo-EM structure of M1R-iperoxo (PDB:6OIJ). (B) X-ray structure of M1R-77-LH-28-1 (PDB:6ZFZ). (C) X-ray structure of M1R-atropine (PDB:6WJC). (D) X-ray structure of M1R-tiotropium (PDB:5CXV). All the potential binding pockets are shown as a meshed-coloured cartoon with views from each ligand.

M1-AChR crystals for the molecular docking study were selected based on their co-crystallized ligands (similar and dissimilar structural compounds). Therefore, the crystal structures of human M1-AChR in complex with two agonists (PDB ID 6OIJ, 6ZFZ) and two antagonists (PDB ID 6WJC, 5CXV) were employed. The crystal structures were chosen according to the ligand size (molecular volume), this is a small and large ligand for the agonists and antagonists. Iperoxo was used as a small agonist (PDB:6OIJ), while 77-LH-28-1 (PDB:6ZFZ) as a large agonist. On the other hand, atropine (PDB:6WJC) was considered a small antagonist compared to tiotropium (PDB:5CXV). The four crystal structures of the M1-AChR (PDB ID 6OIJ, 6ZFZ, 6WJC, 5CXV) were downloaded from Protein Data Bank (PDB).^47–49^ Structural patterns were measured by X-ray diffraction with resolutions of 2.17 Å (ID: 6ZFZ), 2.55 Å (ID: 6WJC), 2.7 Å (ID: 5CXV), and by cryo-electron microscopy with a resolution of 3.3 Å (ID: 6OIJ).^31,49–51^ The PDB files were prepared by deleting all non-protein atoms, including G proteins and other accessory molecules bound to the protein. For each protein, the missing hydrogen atoms were added based on the protonation state of the titratable residues at pH 7. After treatment, the receptors were coupled with their respective ligands. To facilitate the binding site location on which to focus the docking calculations, a simple druggability score is provided for each (sub)pocket at the receptor, established on a linear combination of volume, surface area, and hydrophobicity (ESI, Fig. S1†), solely based on the 3D structure of the muscarinic receptors analysed using DoGSiteScorer Binding site detection from Protein Plus (Fig. 2).

For each crystal, molecular docking was performed targeting the M1-AChR binding site at the co-crystallized ligand binding site. AutoDock Vina 1.1.2 in UCSF Chimera^52^ was used to determine binding modes and binding energies of ligands to the receptor. The AM1-BCC method partial charge scheme was used, with a 15 Å cubic box. The ligands evaluated were docked to the orthosteric binding site of the M1-AChR, which was defined using the co-crystallized ligand. Note that the co-crystallized ligands were removed from the receptor before starting the docking procedure. To validate the docking results, the conformation of each co-crystallized ligand was reproduced with a root-mean-square deviation (RMSD) of 0.82 Å for 6OIJ, 1.2 Å for 6WJC, 0.85 Å for 6ZFZ and 0.29 Å for 5CXV (ESI, Fig. S3–S6†). This allowed us to evaluate the rest of the molecules used in this study, in the same way as the ligands with which the method was validated. The docking results were then analysed, and we were left with the most suitable conformation according to the ligand interaction profile. The best-docked score poses as determined by AutoDock software were selected and visualized using BIOVIA Discovery Studio Visualizer (DSV).^53^ 2D representations of the ligand-M1AChR molecular interactions: hydrophobic, electrostatic, and hydrogen bonds interactions were generated and analysed using DSV to highlight important residues in the protein–ligand interaction of the complex. Finally, we sought to correlate the interaction energy obtained from docking with pKi values, however, QSAR modeling was necessary to validate the molecular docking energy.

### Second QSAR model approximation

In this second approach, an exhaustive calculation of molecular descriptors was performed. The 59 compounds were exported to the online software SwissADME^54^ and ChemDes.^55^ With these servers, several families of 0D–2D molecular descriptors were calculated – which do not depend on the conformation of the molecules – such as those belonging to the family of topological, constitutional, and molecular properties. Also, interaction energy values (*E*_inter_), obtained from molecular docking with M1-AChR, were included in the analysis as a molecular descriptor. Using stepwise regression analysis, more than 300 different descriptors from the training set were plotted (ESI, Fig. S7†). Molecular descriptors with high frequencies were selected and used in the final regression analysis using all ligands from the training set.

For this QSAR study the multiple linear regression (MLR) method was used to construct the QSAR model. MLR is a technique that establishes a linear relationship between a dependent variable Y (pKi values) and several independent variables X (*E*_inter_, and other molecular descriptors). The model is fitted such that the sum of squares of the differences between the observed and predicted values is minimized. MLR estimates the values of the regression coefficients (*R*^2^) by applying the least squares curve fitting method. The model creates a straight-line (linear) relationship that best approximates all individual data points. For this case, the search for the best mathematical model was made by combining the molecular descriptors with the greatest correlation to the potency of the compounds with the binding energy value of each type of compound (agonists/antagonists).

### Statistical validation

The final QSAR models were internally and externally validated. The internal validation procedure used was leave-one-out (LOO) cross-validation (*Q*_LOO_^2^), *R*^2^, *R*_ADJ_^2^, standard deviation, and *F*-function. Where, *R*^2^ is the squared correlation coefficient, *R*_ADJ_^2^ is the adjusted squared correlation coefficient, *s*, is the standard error of the regression and *F* is Fisher's coefficient for the regression. *R*^2^ is a measure of how well the regression line approximates the actual data points. *s*, represents the average distance of the observed values falling from the regression line. Smaller values (*s* = 0.51) are better because they indicate that the observations are closer to the fitted line. The *F*-test reflects the relationship between the variance explained by the model and the variance due to the error in the model. Due to the number of molecules evaluated, the cross-validation of leaving one out of the training set (*Q*_LOO_^2^) describes the stability of the regression model obtained by focusing on the sensitivity of the model to the removal of one of data.

The quality and predictive ability of the QSAR models developed for this case were evaluated using the following statistical measures: *F*-test (Fisher's value) for statistical significance, standard deviation (*s*), coefficient of determination (*R*^2^), adjusted coefficient of determination (*R*_ADJ_^2^), QUIK rule, redundancy rules (RP) and overfitting rules (RN). For the predictive ability of the model, different statistical parameters were considered, such as the correlation coefficient *Q*_LOO_^2^, *Q*_LMO_^2^, *Q*_BOOT_^2^, *Q*_ASYM_^2^, *Q*_EXT_^2^, and *Y*_scrambling_. This strategy was similar to the one used in other systems studied by our group.^56,57^

## Results and discussion

### QSAR study

After the construction and structural optimization of all the compounds in this study, a structural analysis over the compounds and their biological activity over the M1-MAChR (pKi). From this analysis, a correlation of the molecular volume with the pKi of the agonists was found (eqn (1)). Nevertheless, to achieve this the partial agonists were necessary in the mathematical model construction (ESI, Fig. S8 and S9†). We used this approach based on the theory that partial agonists have this type of activity because they can act as agonists or antagonist.

1 pKi = −0.00002[*V*^2^] + 0.02387[*V*] + 1.15472

*Q*_LOO_^2^ = 69.41; *R*_AGONIST_^2^ = 75.3; *s* = 0.769; *F* = 30.5

From the eqn (1) we can see that by increasing the molecular volume of the agonists their potency will increase. Nevertheless, after achieving a molecular volume of 1193.5 Å^3^ the activity of the agonists will decrease, if we only consider this descriptor affecting the pKi of the compounds.

In the same way, we analyzed the antagonist activity based on their molecular volume value. As for the agonists, the correlation was obtained for the antagonists until we considered the partial agonist in the construction of the mathematical model. As for the agonists, the antagonists have a similar correlation based on the molecular volume eqn (2), in which the molecular volume has a limit value, after which the potency of the antagonists will decrease.

2 pKi = −0.00009[*V*^2^] + 0.07305[*V*] − 5.82246

*Q*_LOO_^2^ = 51.03; *R*_ANTAGONIST_^2^ = 61.83; *s* = 1.051; *F* = 23.5

**Fig. 3:**
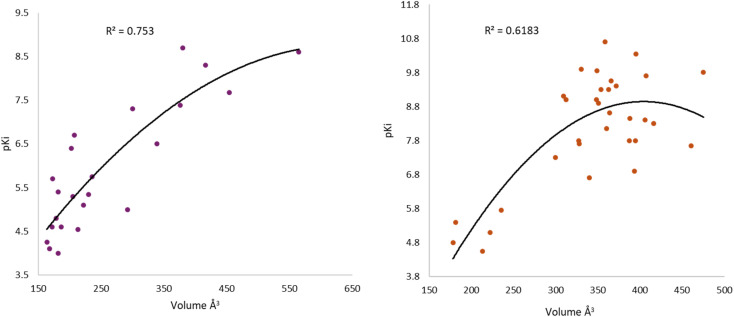
Graphical representation of the quadratic polynomial relation of the potency (pKi) of agonists (left) and antagonists (right) to their molecular volume.

The values of the statistical parameters of eqn (1) and (2) show that a better correlation between the molecular volume and the potency of the compounds is achieved by the agonists than for the antagonists. Also, these correlations are shown in the Fig. 3 graphs.

From this analysis, we were interested to understand the correlation between the pKi with the molecular volume of agonists and antagonists of the M1-MAChR based on the interaction profile of these compounds. Therefore, to achieve this we used the computational molecular docking approach.

### Analysis of M1 MAChR-agonists binding mode

The analysis of different M1R-ligand binding was carried out with the following crystal structures; M1R: iperoxo (PDB code: 6OIJ) complex, M1R: 77-LH-28-1 (PDB code: 6ZFZ) complex, M1R: atropine (PDB code: 6WJC), M1R: tiotropium (PDB code: 5CXV). For comparative analysis of different ligand binding modes of M1-AChR, we used regions of the orthosteric ligand binding site previously defined,^58,59^ including the centrally located amine pocket (D3.32, S3.36, W6.48, Y6.51, Y7.39, Y7.43), connecting the main pocket (Y3.33, N3.37, W4.56, T5.46, N6.52) and minor pocket (F2.56, L2.60, Y2.61, A/W3.28, L3.29), extending into the extracellular vestibule (Y2.64, W23.50, C45-50, I45.52). Most of the amino acids contributing to the orthosteric binding site of muscarinic acetylcholine receptors (mAChR) are in the transmembrane (TM) domain.^35,60,61^ In the orthosteric amine pocket, the four co-crystallized ligands show a conserved binding mode at residues D3.32, W6.48, Y6.51, and Y7.39 (Table 1), corresponding to the ligand contact residues located in TM3, TM6, and TM7 of the predicted M1R.^62^ The key positions in TM3, TM6, and TM7 form a consensus scaffold of the ligand binding pocket, and variation in the amino acids occupying the topologically equivalent positions contributes to ligand specificity at different receptors. Slightly different bindings were observed for the orthosteric binding site depending on the co-crystallized agonist/antagonist. The co-crystallized agonists share most of the conserved residues in the amine pocket and major pocket binding sites, with less interaction of conserved residues in the previously defined minor pocket and extracellular vestibule (Fig. 2, and Table 1).

**Table 1:** Ligand contacting residues in the receptor crystal structure of the agonist/antagonist-bound human M1-receptor cocrystallized. Superscripts indicate Ballesteros-Weinstein numbering

Aminergic GPCR ligand binding site regions	PDB	Generic residue positions					
**Amine pocket**		D^3.32^	S^3.36^	W^6.48^	Y^6.51^	Y^7.39^	Y^7.43^
	6OIJ	Yes	—	Yes	Yes	Yes	Yes
	6ZFZ	Yes	—	Yes	Yes	Yes	Yes
	6WJC	Yes	—	Yes	Yes	Yes	Yes
	5CXV	Yes	Yes	Yes	Yes	Yes	Yes

**Major pocket**		Y^3.33^	N^3.37^	W^4.57^	T^5.46^	N^6.52^	
	6OIJ	Yes	—	Yes	Yes	Yes	
	6ZFZ	Yes	—	Yes	Yes	Yes	
	6WJC	Yes	Yes	Yes	—	Yes	
	5CXV	Yes	Yes	Yes	—	Yes	

**Minor pocket**		F^2.56^	L^2.60^	Y^2.61^	A/W^3.28^	L^3.29^	
	6OIJ	—	—	—	—	—	
	6ZFZ	—	—	Yes	Yes	Yes	
	6WJC	—	—	—	—	—	
	5CXV	—	—	—	—	—	

**Extracellular vestibule**		Y^2.64^	W^23.50^	C^45.50^	I^45.52^		
	6OIJ	—	—	—	—		
	6ZFZ	Yes	Yes	Yes	—		
	6WJC	—	—	—	—		
	5CXV	—	—	—	—		

In TM6, residues W6.48 and Y6.51 make an important non-covalent contact for the conserved structural scaffold. W6.48 is a key residue for receptor activation as well as binding.^63^ Mutation of the conserved W6.48 residue detrimentally decreases the binding affinity of several ligands, without affecting the affinity of allosteric modulators, as they are not targeted to the main binding pocket.^64–66^ In the main pocket, the co-crystallized agonists/antagonists contact the Y3.33 residue located in TM3, which is also part of the key residues of the ligand binding pocket. Mutation of Y3.33A, Y6.51A greatly impairs orthosteric ligand binding, decreasing or abolishing binding of the tested ligand, without affecting binding of allosteric modulators.^67–69^

The butyl residue of 77-LH-28-1 is flexible and adopts alternative binding modes between Y3.33/W4.57 in the main pocket, which facilitates its entry into the recognition pocket.^49^ W4.57 only makes van der Waals contact with iperoxo, resulting in a lower affinity for M1-AChR relative to 77-LH-28-1 (ESI, Fig. S2–S10†). T5.46A mutation does not affect the binding affinities of antagonists but significantly modifies the binding affinity for agonists through loss of H-bond interactions and changes in binding mode.^70^ This indicates that T5.46 is not involved in antagonist binding (Table 1).

**Fig. 4:**
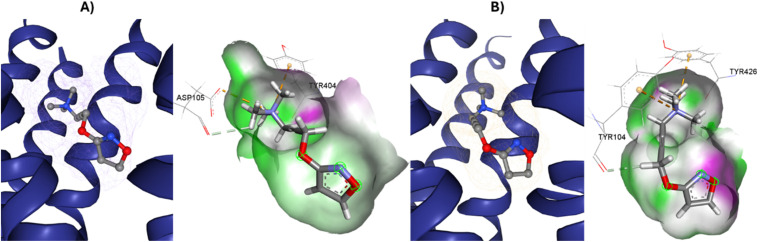
Variation of muscarinic receptor binding cavity volume. (A) Cryo-EM structure of M1R-iperoxo (PDB:6OIJ), accessible volume 481.44 Å^3^. (B) X-ray structure of M2R-iperoxo (PDB:4MQS), accessible volume 391.02 Å^3^.

Iperoxo is the most potent and effective muscarinic agonist, which has served as an orthosteric moiety for other bitopic or dualsteric ligands.^71–73^ Iperoxo is a highly effective agonist for both M1R and M2R, but its affinity for M2R is higher than for M1R.^74^ Although M1R and M2R dock to different G proteins, the overall structure of active M1R is like that of the active M2R conformation.^51^ The conformations of residues critical for receptor activation such as D3.49 R3.50 Y 3.51 N7.49 P7.50 xxY7.53, and the P5.50 I/V3.40 F6.44 motifs,^59^ are also similar between the active conformations of M1R and M2R, suggesting that the activation mechanism is shared between M1R and M2R. Although the iperoxo-coordinating residues are identical and the side-chain conformations between M1R and M2R are similar,^75^ the accessible volume from the surface of the orthosteric binding pocket in M1R, 481.44 Å^3^, is significantly larger than that of M2R, 391.02 Å^3^ (ESI, Fig. S1†). This could contribute to the lower affinity of iperoxo for M1R compared to M2R (Fig. 4).

**Fig. 5:**
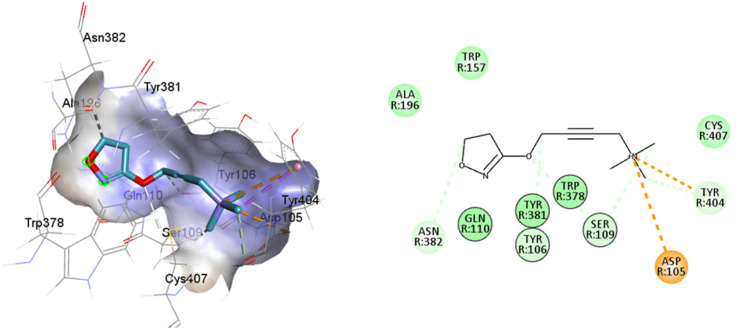
2D and 3D interaction pose of iperoxo docked at the binding pocket of 6OIJ with their respective interacting residues. Green lines: hydrogen bonds; green intense residue: conventional hydrogen bond; remarkable residue: hydrophobic interactions; orange: pi–cation; pink: pi–pi T-shaped or pi–pi stacked; orange: salt bridge or cation–pi interaction; purple: alkyl and pi–alkyl.

There is a significant difference in the major pocket of the iperoxo binding site when comparing the cryo-EM structure of M1R-iperoxo-Gq/11 (M1R-ip-Gq/11) to the X-ray structure of M2R-iperoxo-Gi (M2R-ip-Gi). N3.37 and Y3.33, interact with iperoxo in TM3,^62^ however, N3.37 is absent in M1R-ip-Gq/11, decreasing M1R/iperoxo binding affinity. Nevertheless, regardless of the type of structural identification method and muscarinic receptor subtype, the iperoxo binding site is very similar due to high sequence identity in the orthosteric pocket between the subtypes. Conserved N6.52 residue is one of the crucial residues for ligand binding in muscarinic acetylcholine receptors, and its mutation decreases or abolishes ligand binding, creating an unfavourable H-bond geometry between highly conserved residues in the receptor core, the integrity of which is crucial for receptor activation.^70^ N6.52 forms an H-bond with the ester and tertiary alcohol groups of tiotropium, and an H-bond only with the atropine ester group (Fig. 1 and Table 1). In the main pocket, residue N6.52 forms characteristic concerted H-bonds with the amide of 77-LH-28-1 and the isoxazoline cycle of iperoxo (Fig. 1 and Table 1). In this regard, in a small interaction cavity, a small molecule such as iperoxo can interact with the important residues of the orthosteric amine pocket, without interacting with the residues of the minor pocket and the extracellular vestibule (Fig. 5 and Table 1).

**Fig. 6:**
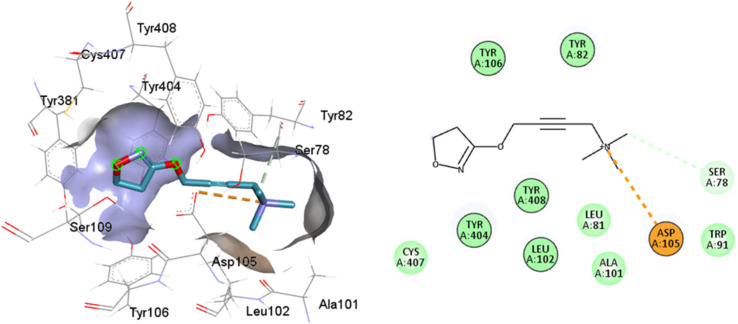
2D and 3D interaction pose of iperoxo docked at the binding pocket of 6ZFZ with their respective interacting residues. Green lines: hydrogen bonds; green intense residue: conventional hydrogen bond; remarkable residue: hydrophobic interactions; orange: pi–cation; pink: pi–pi T-shaped or pi–pi stacked; orange: salt bridge or cation–pi interaction; purple: alkyl and pi–alkyl.

Such interactions are closely related to the ligand volume (ESI, Fig. S1†). The small volume of iperoxo does not allow interaction with all binding pockets of the M1AChR. The conformation obtained from the docking calculation is favorable for iperoxo when using a crystal with a large cocrystallized ligand. In a large volume binding cavity (Fig. 6), iperoxo retains only interaction with D3.32, and binding modes with residues W6.48, Y6.51, and Y7.39 are lost, but it does interact with residues Y2.61, A3.28, L3.29, located in the minor pocket and with W23.50 in the extracellular vestibule (Table 1).

**Fig. 7:**
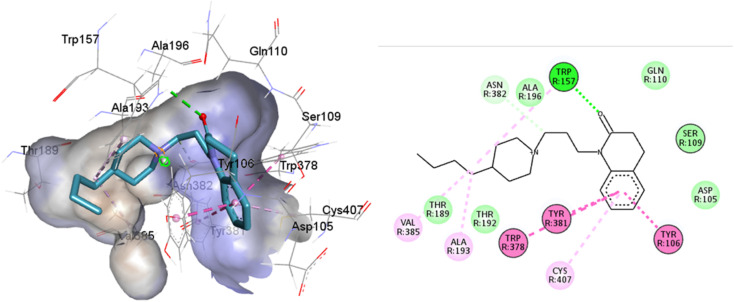
2D and 3D interaction pose of 77-LH-28-1 docked at the binding pocket of 6OIJ with their respective interacting residues. Green lines: hydrogen bonds; green intense residue: conventional hydrogen bond; remarkable residue: hydrophobic interactions; orange: pi–cation; pink: pi–pi T-shaped or pi–pi stacked; orange: salt bridge or cation–pi interaction; purple: alkyl and pi–alkyl.

77-LH-28-1 is a selective M1-AChR agonist that acts through a bitopic mechanism, as it targets the orthosteric and allosteric binding sites simultaneously. In the minor pocket, AC-42 analogues, such as 77-LH-28-1 (PDB:6ZFZ), can interact with the W3.28 residue of M1-AChR through charge–charge interaction from the protonated nitrogen within the piperidine fragment at the centre of the ligand to the negatively charged aspartate side chain (D3.32).^30^ The dihydroquinolinone ring of 77-LH-28-1 forms a complex between the aromatic ring of Y2.64 and the side chain of L3.29, by the positioning of the positively charged piperidine nitrogen near the carboxylate group of D3.32 and the hydroxyl group of Y7.43.^76^ The piperidine ring system cleaves the tyrosine cap. Towards the apical side of the receptor, the tetrahydroquinoline moiety of 77-LH-28-1 establishes hydrophobic contacts with W23.50, L3.29, Y2.64, and Y2.61, as well as with the disulfide bridge between the cysteine residues C3.25-C17845.50, thus binding TM3 to extracellular loop 2 (ECL2). In addition, the tetrahydroquinoline oxygen further forms a water-mediated hydrogen bond with the hydroxyl group of Y2.61.^30^ In TM2, Y2.61 and Y2.64, play a pivotal role in the binding of allosteric compounds in the extracellular vestibule to the orthosteric pocket.^77,78^ The reduced efficacy of 77-LH-28-1 in TM2 mutants indicates that this region is required for M1-AChR activation, suggesting a different role for TM2 in bitopic ligand efficacy compared to orthosteric ligands.^79^ Combined mutations at residues in orthosteric (W6.48, D3.32, Y3.33, Y6.51) and allosteric (Y2.61, Y2.64, Y45.51, W7.34, E/T7.35) sites confirm that 77-LH-28-1 interacts with both binding pockets.^80,81^ However, 77-LH-28-1, a large molecule in a small interaction pocket loses contact with residues D3.32 and Y7.39 and modifies the type of interaction with residues W6.48, Y6.51, to a less favourable pi–pi T-shaped configuration (Fig. 7). Furthermore, 77-LH-28-1, loses contact with all conserved residues in the minor pocket and extracellular vestibule (Table 1).

**Fig. 8:**
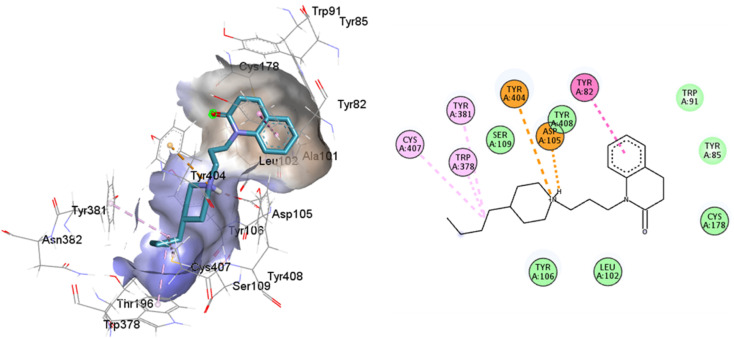
2D and 3D interaction pose of 77-77-LH-28-1 docked at the binding pocket of 6ZFZ with their respective interacting residues. Green lines: hydrogen bonds; green intense residue: conventional hydrogen bond; remarkable residue: hydrophobic interactions; orange: pi–cation; pink: pi–pi T-shaped or pi–pi stacked; orange: salt bridge or cation–pi interaction; purple: alkyl and pi–alkyl.

An oversized ligand such as 77-LH-28-1 does not perform well in a crystal with a small co-crystallized ligand, because interactions are deformed and contacts with key residues for agonism are lost (Fig. 8). An increase in the interaction cavity of the crystal, related to the ligand volume, promotes 77-LH-28-1 to interact with residues located in the minor pocket and the extracellular vestibule of the M1AChR (Fig. 8), improving the affinity of the ligand for the receptor (ESI, Fig. S2†).

### Analysis of M1 MAChR-antagonists binding mode

**Fig. 9:**
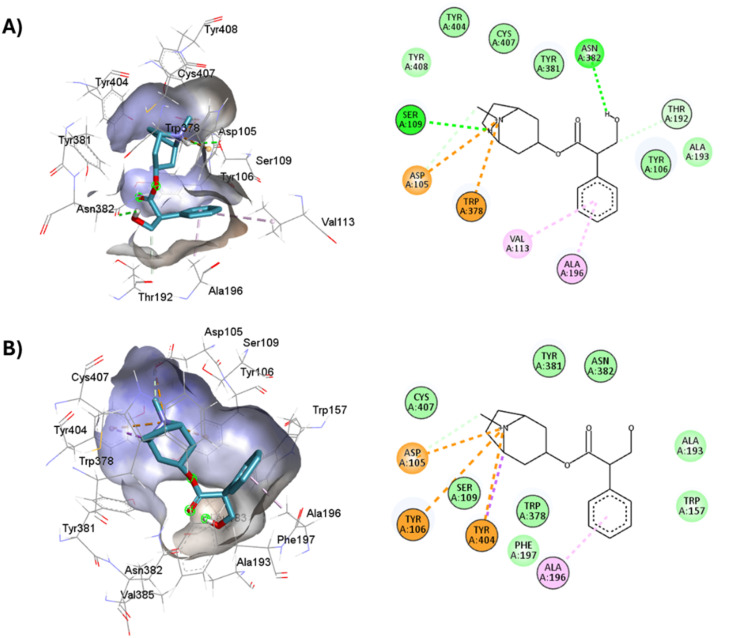
2D and 3D representation of the docked antagonists into the binding site of different crystal structures. Interaction poses of atropine docked at the binding pocket of (A) 6WJC and (B) 5CXV with their respective interacting residues. Green lines: hydrogen bonds; green intense residue: conventional hydrogen bond; remarkable residue: hydrophobic interactions; orange: pi–cation or pi–sulfur; purple: pi–sigma; pink: pi–alkyl.

Full agonists can stimulate maximal GPCR activity inducing maximal responses. Partial agonists trigger a smaller response than a full agonist and act as a type of antagonist in the presence of full agonists. Typically, antagonists are larger than agonists, therefore, they block agonist-stimulated responses. Unlike agonists, antagonists have the characteristic of blocking the response by increasing the size of the molecule while maintaining minimal interactions with the orthosteric amine pocket and the major pocket, without contacting the residues located in the minor pocket and the extracellular vestibule of the M1AChR (Table 1). D3.32 is a highly conserved residue in aminergic receptors and is surrounded by an aromatic cage consisting of Y3.33, W6.48, Y6.51, Y7.39 and Y7.43.^82^ These five aromatic residues, together with W4.57, Y6.51 (Table 1), generate hydrophobic interactions with the tropane group of atropine and tiotropium (Fig. 1). Mutation of these hydrophobic residues significantly affects ligand binding.^70^ Concerning ligand volume, most of the antagonists evaluated showed a positive similarity in volume difference when using a larger ligand such as tiotropium (ESI, Fig. S1†). The difference in the volume of smaller ligands such as atropine is negative to the other molecules evaluated (ESI, Fig. S1†). The conformation of low-volume atropine results in a limited interaction at the receptor binding site (Fig. 9) with fewer hydrophobic contacts than a larger molecule such as tiotropium.

**Fig. 10:**
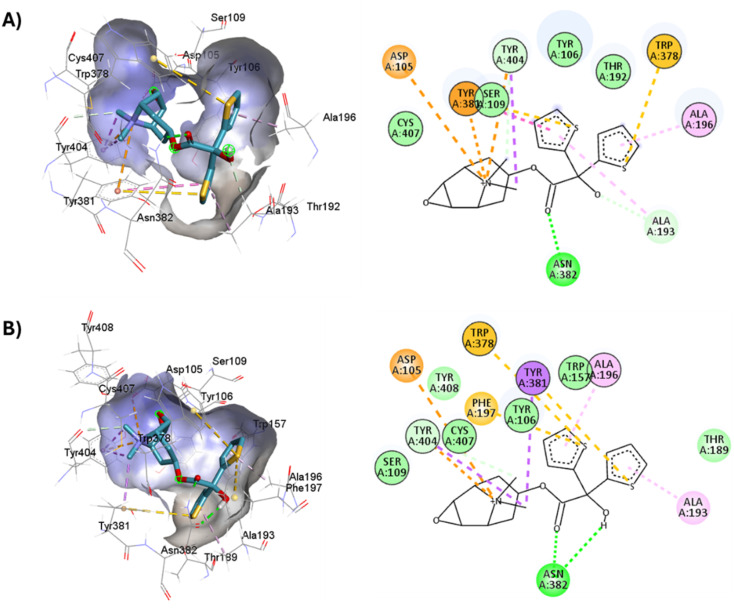
2D and 3D representation of the docked antagonists into the binding site of different crystal structures. Interaction poses of tiotropium docked at the binding pocket of (A) 6WJC and (B) 5CXV with their respective interacting residues. Green lines: hydrogen bonds; green intense residue: conventional hydrogen bond; remarkable residue: hydrophobic interactions; orange: pi–cation or pi–sulfur; purple: pi–sigma; pink: pi–alkyl.

D3.32 forms an ionic interaction, while residues Y3.33, W6.48, Y7.39, Y7.43 form pi–cation interactions with the conserved tertiary and quaternary amine moieties of M1-AChR ligands according to mutation studies and ligand structure–activity relationship analysis.^58^ Mutation at the conserved D3.32E residue significantly decreases the M1-AChR binding affinity of ligands with quaternary amines in their structure, without significantly affecting the affinity of ligands possessing tertiary amines.^78,81,83,84^ The ionic character of the carboxylate group and the size of the side chain of D3.32 are important for ligand binding to quaternary amines, such as iperoxo and tiotropium, not so for 77-LH-28-1 and atropine, as they possess tertiary amines (Fig. 1). Due to the different sizes of the antagonists, atropine, and tiotropium, a slight inward shift of TM5 has been observed in the atropine-bound M1-AChR compared to the tiotropium-bound structure.^50^ Atropine has a single phenyl group facing TM3 and TM4, whereas tiotropium has two thienyl groups (Fig. 1), with the second thienyl facing TM5. TM3 is key for ligand binding, and the portion toward the cytoplasm makes contacts with TM5 and TM2, and likely, the lack of the second ring in atropine (Fig. 1) contributes to a lower affinity (ESI, Fig. S2†). A bulky ligand, such as tiotropium, induces a better conformational rearrangement at the receptor binding site (Fig. 10), resulting in greater binding site specificity and therefore better binding affinity (ESI, Fig. S1†).

S3.36 residue makes consensus contact with various ligands in class A GPCRs.^62^ Mutation of S3.36 residue affects ligand binding affinity.^85^ S3.36 forms an H-bond with the tiotropium epoxide residue (Fig. 1). This interaction confers an increased M1R/tiotropium binding affinity, on the other co-crystallized ligands used in this work (ESI, Fig. S2†). Mutation of N3.37 decreases the binding of acetylcholine, QNB, and pirenzepine.^31^ Interestingly, the N3.37 residue, which is also part of the key scaffold of the ligand-binding pocket, interacts only with atropine and tiotropium, which possess ester groups in their structure (Fig. 1), absent in the agonists, enabling H-bonds and/or hydrophobic interactions between N3.37 and the ester moiety (Table 1). Muscarinic receptors possess a large extracellular vestibule containing residues that contribute to an allosteric site.^86–88^ A series of tyrosine residues form an aromatic “cap” that restricts the dissociation of co-crystallized antagonists, preventing direct interaction with residues in the minor pocket and extracellular vestibule (Table 1). The residues that form the tyrosine cap involve residues Y3.33, Y6.51, Y7.39, and Y7.43.^89^ These tyrosine residues come together to form a lid over the orthosteric binding pocket and separate it from the allosteric pocket.^32^ These residues have been implicated in the regulation of antagonist dissociation from the orthosteric binding site.^66^ In molecular dynamics simulations, these tyrosine's have been observed to change their rotameric state, and their flexibility also depends on the quality of the polar interactions formed by the ligands with residue N6.52.^66,75,90,91^ In the functions predicted by M1-AChR-based molecular modeling studies, N6.52 was found to be critical for antagonist binding, but less involved in agonist binding and receptor activation.^70,90^ The interaction of the double hydrogen bond of N6.52 with tiotropium has a crucial influence on dissociation kinetics,^66^ and together with the tyrosine cap, keeps the antagonists, atropine, and tiotropium, in the orthosteric binding pocket (Table 1).

### Molecular docking analysis

To understand the binding interactions between M1-AChR and other compounds, molecular modeling studies were carried out by docking 59 structurally different ligands into the binding pocket containing the critical residues of the M1 receptor. The binding energies and the resulting different interactions between the ligands evaluated and the receptor are summarized in ESI, Fig. S2.†

**Fig. 11:**
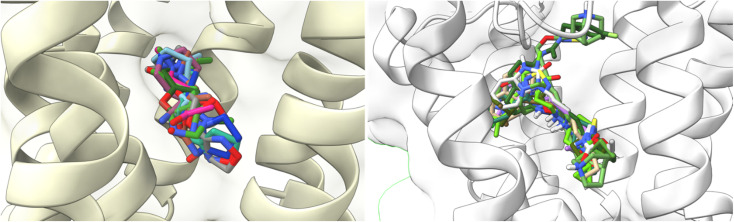
Docking results of the binding mode of small agonists of the M1-AChR in the 6OIJ crystal (left). Binding mode of large agonists of the M1 AChR in the 6ZFZ crystal (right).

The docking scores have a structural interpretation in terms of predicted binding affinity.^92^ In this regard, ligand docking values were modified as a function of the size volume/surface area of ligand interaction at the receptor binding site (Fig. 11; ESI, Fig. S1†).

Both agonists and antagonists showed a decrease in binding energies when the larger ligands were coupled to the reduced cavities (ESI, Fig. S2†). However, the two larger ligands, 77-LH-28-1, and tiotropium, exhibited distinct binding patterns and differed in their binding energies when coupled to more limited cavities. The binding capacity of the agonist 77-LH-28-1, in contrast to iperoxo, is good due to all the pi-type interactions (Table 2), and the binding energy of the resulting complex was −10.1 kcal mol^−1^ (6ZFZ, ESI, Fig. S2†). The binding capacity of 77-LH-28-1 was reduced when coupled to a smaller interaction cavity, with a resulting binding energy of −5 kcal mol^−1^ (6OIJ), indicating less strong ligand binding (ESI, Fig. S2†).

**Table 2:** Interaction types and amino acids involved in the agonism (iperoxo, 77-LH-28-1) or antagonism (atropine, tiotropium) at M1-AChR. All graphical presentations of the docked complexes were analysed using BIOVIA discovery studio visualizer

Ligand	Hydrogen bond (HB) interaction	Bond length (Å) for HB interaction	Hydrophobic interaction	Pi–cation interaction	Pi–sigma interaction	Pi–alkyl interaction	Pi–sulfur interaction
Iperoxo	Ser109	2.9	Tyr404	Tyr404 (4.4 Å)	Tyr404 (3.5 Å)	—	—
			Tyr106				
			Tyr381				
			Trp378				
77-LH-28-1	Asp105	1.9	Cys407	Tyr404 (4.2 Å)	—	Tyr381 (4.8 Å)	—
			Ser109			Trp378 (4.6 Å)	
			Tyr82				
			Tyr106				
			Tyr381				
			Tyr404				
			Trp378				
Atropine	Asn382	2.4	Ala196	Trp378 (4.3 Å)	—	Ala196 (3.7 Å)	—
	Ser109	3.1	Ser109			Val113 (5.4 Å)	
			Tyr106				
			Tyr381				
			Tyr404				
			Val113				
Tiotropium	Asn382	2	Ala193	Tyr404 (4 Å)	Tyr381 (3.7 Å)	Ala193 (4.6 Å)	Phe197 (5.9 Å)
			Ala196		Tyr404 (3.4 Å)	Ala196 (3.6 Å)	Tyr381 (5.3 Å)
			Tyr106				Trp378 (5 Å)
			Tyr381				
			Tyr404				

The conformational energy of 77-LH-28-1 was minimized by a larger H-bond spacing, by the absence of the electrostatic pi–cation interaction, and by an increase in hydrophobic interactions with nonpolar amino acids (Table 2). The plasticity of the binding cavity led to changes in the interaction scaffold, and modifications in the binding energy of 77-LH-28-1 (6OIJ), were compensated through non-covalent pi–pi stacked (Tyr106) and pi–pi T-shaped (Tyr381, Trp378) interactions (Table 2; ESI, Fig. S10).† Unlike 77-LH-28-1, iperoxo, regardless of the size of the receptor interaction cavity, and due to its structure and size, did not show significant changes in binding energies (6OIJ: −6.1 kcal mol^−1^ and 6ZFZ: −5.9 kcal mol^−1^, ESI, Fig. S2†), most likely due to the loss of pi–cation and pi–sigma interactions (Table 2).

**Fig. 12:**
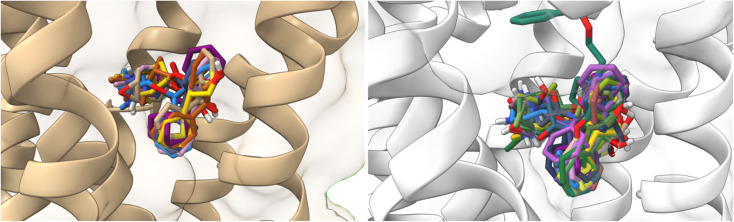
Docking results of the binding mode of small antagonists of the M1-AChR in the 5CXV crystal (left). Binding mode of small antagonists of the M1-AChR in the 6WJC crystal (right).

On the other hand, atropine and tiotropium binding energies changed in a size-dependent manner of the interaction cavity (Fig. 12).

Tiotropium exhibits a significant number of interactions with receptor, which confers a high affinity for it, and a greater binding energy of −9.8 kcal mol^−1^ (5CXV, ESI, Fig. S2†). In a reduced volume of the binding site, the affinity energy of tiotropium modified to −8.8 kcal mol^−1^ (6WJC), with important changes determined by an increased H-bond spacing, a decrease in pi–sulfur interactions, and an increase in hydrophobic interactions with nonpolar amino acids (Table 2). These modifications in the binding energy of tiotropium were buffered by an increase in pi–cation interactions and the appearance of pi–pi stacked (Trp157) and pi–pi T-shaped (Tyr381) interactions (Table 2; ESI, Fig. S10†). The docking energy of atropine resulted in more negative binding energies, from −8.3 kcal mol^−1^ (6WJC) to −8.9 kcal mol^−1^ (5CXV), suggesting a stronger ligand binding (ESI, Fig. S2†). An increase in the volume of the binding site allowed atropine to increase the number of pi–cation interactions, forming pi–sigma interactions, despite making fewer hydrophobic contacts and having fewer pi–alkyl interactions (Table 2). Molecular docking of larger ligands, such as the agonist 77-LH-28-1 and the antagonist tiotropium, into smaller cavities (6OIJ and 6WJC, see ESI, Fig. S1†) sterically prevents the correct assembly of interactions within the ligand-binding cavity. The cavity plasticity, and the structure of these ligands, allow them to generate a stacked arrangement of aromatic pi–pi interactions, as there is steric repulsion that misrepresents the nature of these interactions at typical pi and CH–pi stacking distances, where charge penetration is significant.^93^ Furthermore, pi–pi stacking generates a relatively small binding energy,^94^ so the formation of pi–pi interactions (Table 2) was not sufficient to generate an enhanced binding energy (ESI, Fig. S2†). These results suggest that the generation of pi–pi interactions of 77-LH-28-1 and tiotropium in a smaller binding cavity enhances charge dispersion due to the quadrupole electrostatic interactions characteristic of pi–pi interactions.^93^

### Ligand intermolecular interactions analysis at the orthosteric binding site based on coupling energies

**Fig. 13:**
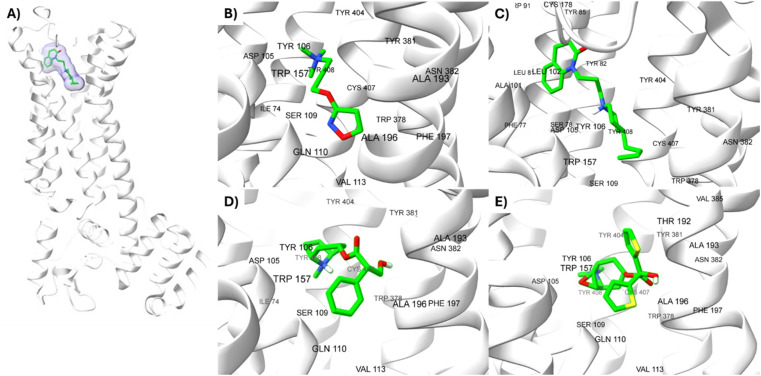
Structures of M1AChR bound to different ligands. The structures of the agonists/antagonists bound M1receptor are highly like each other in overall fold. (A) For the sake of clarity, only of the ligand 77-77-LH-28-1-bound to M1 receptor is shown. (B–E) Orthosteric ligand binding sites (green-coloured) observed in the crystal structure in complex with the agonist/antagonist-bound [(B) iperoxo, (C) 77-77-LH-28-1, (D) atropine, (E) tiotropium].

The lowest energy pose selected for each ligand-receptor complex was analysed using BIOVIA DSV. The binding sites of the experimental ligands in the M1-AChR/ligand complex are shown in Fig. 13. The tyrosine residues Y7.39 and Y7.43, together with the orthosteric site residues W6.48, D3.32, Y3.33, and Y6.51, were used as a reference in molecular modeling to characterize the crystal bound to its ligands (agonists or antagonists) and to determine the similarity between the obtained energy values compared to the experimental data for each crystal.

**Fig. 14:**
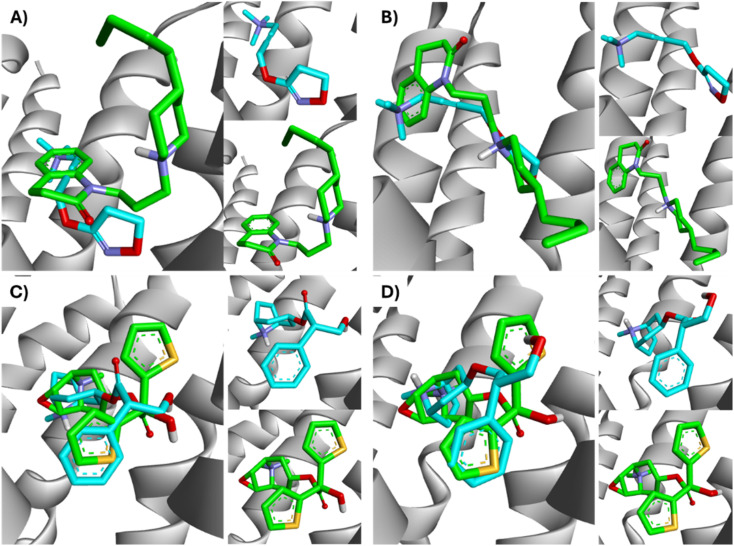
Superimposition of the best-docked M1-AChR/ligands complexes. (A) Comparison of top-ranked pose obtained from molecular docking of 77-LH-28-1 (green) with the crystallographic binding mode of iperoxo (cyan) complex (PDB:6OIJ), and (B) iperoxo with the crystallographic binding mode of 77-LH-28-1 complex (PDB:6ZFZ). (C) Comparison of top-ranked pose obtained from molecular docking of tiotropium (green) with the crystallographic binding mode of atropine (cyan) complex (PDB:6WJC), and (D) atropine with the crystallographic binding mode of tiotropium complex (PDB:5CXV).

All ligands are in the substrate binding region. As shown in Table 1 and Fig. 5–10, all four ligands show contacts with key residues in the centrally located amine pocket as well as interactions with residues in the main pocket. The large agonist binding such as 77-LH-28-1 (ESI, Fig. S2†), in a reduced interaction pocket, modifies the ligand conformation and thus the interactions with the receptor (Fig. 14B). Not so for a small-volume agonist such as iperoxo (Fig. 14A). However, regardless of the size of the crystal interaction cavity, the conformation of the antagonists is not modified due to the ligand volume and binding site volume increase (Fig. 14C and D) (ESI, Fig. S2†).

**Fig. 15:**
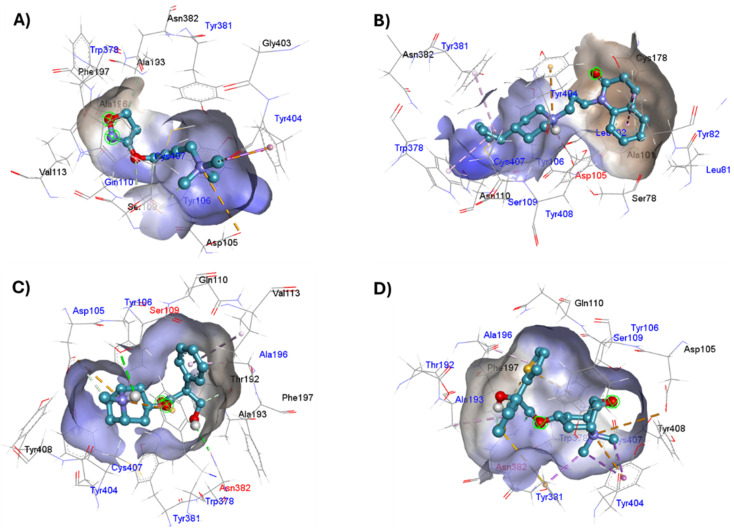
Binding modes of M1AChR ligands. Cartoon representation of the interactions of iperoxo (A), 77-LH-28-1 (B), atropine (C), and tiotropium (D) at the binding pocket of M1AChR. Three-dimensional diagrams describing essential interactions with residues that are crucial for the activation/inactivation of M1AChR are presented. Three-dimensional diagram describing ligands interactions by the conventional hydrogen bonds formation red-coloured and hydrophobic interactions blue-coloured at M1AChR.

The four co-crystallized ligands make hydrophobic contact with Y3.33 and Y6.51 (Fig. 15, ESI, Fig. S10†). In addition, Y6.51 forms pi–alkyl interactions on the aromatic group and the alkyl group of 77-LH-28-1, and pi–sulfur with the thienyl group of tiotropium. For its part, Y7.39 makes electrostatic pi–cation interactions with iperoxo, 77-LH-28-1, and tiotropium, except for atropine which makes pi–cation interaction with W6.48 (Fig. 15, ESI, Fig. S10†). Unlike interactions with tyrosine residues, interactions with W6.48, D3.32 are more heterogeneous. W6.48 forms hydrophobic interactions with iperoxo, pi–alkyl with 77-LH-28-1, pi–cation with the tropane group of atropine, and pi–sulfur with the thienyl group of tiotropium. While D3.32, forms H-bond with iperoxo and tiotropium, a 77-LH-28-1 salt bridge, and an electrostatic interaction with atropine (Fig. 15, ESI, Fig. S10†). However, highly polar sites are less likely to bind.^95^ In this context, the number of amino acids involved in hydrophobic interaction with iperoxo (Tyr404, Ty106, Tyr381, Trp378), 77-LH-28-1 (Cys407, Ser109, Trp378, Tyr82, Tyr106, Tyr381, Tyr404), atropine (Ala196, Ser109, Tyr106, Tyr381, Tyr404, Val113), and tiotropium (Ala193, Ala196, Tyr106, Tyr381, Tyr404) (Fig. 15), shows an inverse relationship between hydrophobic pockets and *D* score values (ESI, Fig. S1†). As the number of hydrophobic contacts increases, the *D* score, and thus the affinity of the ligand for the receptor, decreases.

A binding site is not necessarily “druggable” simply because it binds a ligand; the ligand needs to have other properties as well, such as non-covalent interactions involving pi systems that are fundamental to biological events such as protein–ligand recognition. Pi–cation interactions play an important role in the recognition of common cationic functional groups within ligands by specific aromatic residues within the protein.^96^

The physicochemical characteristics of pi–cation interactions are particularly well suited to the dual hydrophobic/hydrophilic environment of membrane proteins.^97^ Pi–cation electrostatic interactions preferentially occur through the aromatic side chains of tyrosine (Tyr, Y) or tryptophan (Trp, W). Iperoxo, 77-LH-28-1, and tiotropium interact with the phenolic ring of the Y7.39 residue, whereas atropine interacts with the indole group of W6.48 (Table 2). Unlike the phenol ring of Tyr, the indole ring of Trp allows it to contact a larger number of cations. This confers a higher affinity of atropine for M1-AChR than iperoxo or 77-LH-28-1, but no higher than tiotropium (Fig. 1; ESI, Fig. S2†). For small-volume ligands, such as iperoxo, pi–cation interactions between its trimethylammonium group and aromatic residues of Tyr are critical for recognition at the ligand binding site.^75^ Furthermore, iperoxo, along with tiotropium, are the only ligands in this study capable of both pi–cation and pi–sigma interactions at the same time, with a shorter pi–sigma interaction distance, in contrast to pi–cation in both ligands (Table 2, ESI, Fig. S2†). Pi–sigma interactions result in a lower energy bond orbital, and, therefore, the bond is more stable compared to pi–cation interactions.^98^ The presence of both types of interaction in these two ligands reflects an expansion of possible binding sites and greater flexibility in non-covalent binding to nucleophiles. However, tiotropium, in addition to forming pi–sigma bonds with Y7.39, also does so with Y6.51 (Table 2), allowing it better binding flexibility, and, consequently, better affinity for M1-AChR.

Other interactions that were observed within the crystal complexes include pi–alkyl interactions, which help to enhance the hydrophobic interaction of the ligand in the receptor binding pocket. 77-LH-28-1, atropine and tiotropium showed pi–alkyl interactions (Table 2), except iperoxo due to its size and the number of hydrophobic interactions (Fig. 5–8). The geometric preference of interaction between the alkyl groups and the aliphatic group of Ala196 in the antagonists was found to be very similar (3.6 Å and *θ* = 0°) to the previously reported preferred configuration (*R* = 3.7 Å and *θ* = 0°),^99^ unlike Ala193, Trp378, Tyr381, and Val113 residues (Table 2, ESI, Fig. S10†). In addition, pi–sulfur interactions contribute between 0.5 and 2 kcal mol^−1^ in protein binding and stabilization.^100^ Of the ligands analysed, tiotropium is the only one that makes pi–sulfur interaction (Table 2). Two-dimensional comparison of the pi–sulfur interaction geometry and the aromatic groups of residues Phe197, Tyr381, and Trp378, show an in-plane configuration with a separation of ∼5 Å (Table 2, ESI, Fig. S10†), as previously reported.^101^ The unique stability of the iperoxo binding site can be attributed to the large number of pi interactions it promotes, such as electrostatic (pi–cation), hydrophobic (pi–sigma, pi–alkyl) and finally pi–sulfur interactions (Fig. 5–8 and Table 2). The affinity of the ligands (tiotropium > atropine > 77-LH-28-1 > iperoxo) is largely determined by the volume (ESI, Fig. S1†) and the amount of pi interactions. This binding affinity is related to the presence of H-bonds with the amino acids Ser109 [iperoxo (2.9 Å)], Asp105 [77-LH-28-1 (1.9 Å)], Asn382 [atropine (2.4 Å) and tiotropium (2 Å)] (Table 2). The large relative number of aromatic amino acids in the ligand binding pockets and the presence of aliphatic groups involved in aromatic M1-AChR-ligand interactions, tune the molecular recognition events, enhancing binding affinity, hydrophobicity, ligand specificity, and stability.

### QSAR modelling of M1-AChR agonists

Based on the docking results, the M1-AChR agonists were subjected to a QSAR study, combining molecular descriptors of these compounds, of the topological, structural, and molecular properties type with their interaction energy obtained from docking with the M1-AChR conformation of the 6ZFZ crystal for the construction of the mathematical model (Table 3). After the selection of the descriptors, multiple linear regression analysis was performed to generate suitable models that would allow us to categorize the biological activity of the data set.

**Table 3:** Values of molecular descriptors used in the QSAR models and pKi. Fraction of sp^3^ hybridized carbons (*f*Csp^3^), lipophilicity (log*P*)

	Agonist	Molecular descriptors				Antagonist	Molecular descriptors			
	Molecule	E-6ZFZ	NRB	ilog*P*	pKi	Molecule	E-5CXV	fCsp^3^	Mlog*P*	pKi
Partial agonist	AZD6088	−11.3	5	3.73	8.3	QNB	−10.7	0.38	2.69	10.7
	SPP1	−9.6	5	4.15	7.67	Tiotropium	−9.8	0.53	−2.19	10.34
	Xanomeline	−8	7	3.83	7.3	Aclidinium	−8	0.42	−0.65	10.2
	Sabcomeline	−6.7	2	2.24	6.7	*N*-Methyl scopolamine	−9.7	0.61	−2.26	9.9
	LY593093	−11.8	8	4.27	6.2	Glycopyrrolate	−10.1	0.63	−0.86	9.85
	Oxotremorine	−7	2	2.7	5.75	Umeclidinium	−9.4	0.38	0.54	9.8
	(−)-Aceclidine	−6.5	2	1.94	5.4	Propantheline	−10.2	0.43	−0.02	9.7
	Pilocarpine	−6.8	3	1.71	5.1	Ipratropium	−9.4	0.65	−0.97	9.55
	McN-A-343	−8	5	−4.32	5	Revefenacin	−10.4	0.4	2.84	9.4
	Milameline	−5.8	2	2.39	4.8	Telenzepine	−7.8	0.37	2.26	9.4
	HTL9936	−9.2	7	3.94	4.7	4-DAMP	−10.8	0.38	0.29	9.3
	(−)-YM796	−6.9	0	2.69	4.55	Biperiden	−10	0.62	3.86	9.3
Full agonist	NNC 11-1585	−9.6	3	3.71	9.9	Atropine	−8.9	0.59	2.02	9.1
	77-LH-28-1	−10.1	7	4.03	8.7	Benzatropine	−10.3	0.43	3.69	9
	NNC 11-1607	−11.9	6	5.62	8.6	Scopolamine	−9.6	0.59	1.19	9
	Pentylthio-TZTP	−7.4	6	3.68	8.6	Trihexyphenidyl	−10	0.7	3.73	8.9
	Iperoxo	−5.9	7	−0.92	7.89	Dicyclomine	−8.7	0.95	3.63	8.61
	AC-260584	−9.5	7	4.04	7.39	Tolterodine	−9.6	0.45	4.57	8.5
	GSK-1034702	−9.6	2	3.2	6.5	Oxybutynin	−8.7	0.59	3.07	8.45
	Aracaide propargyl ester	−6.3	3	2.58	6.4	Pirenzepine	−8.6	0.32	1.83	8.15
	AC-42	−9.4	8	3.68	6.2	Amitriptyline	−10.3	0.3	4.31	7.8
	Arecoline	−5.9	2	2.26	5.7	Solifenacin	−8.9	0.43	3.53	7.8
	Oxotremorine-M	−6.1	2	−1.2	5.35	VU0255035	−8.7	0.33	−0.42	7.8
	Cevimeline	−7	0	2.42	5.3	Dosulepin	−8.8	0.26	4.36	7.7
	HTL9936	−9.2	7	3.94	4.7	Darifenacin	−9.1	0.32	3.78	7.65
	Acetylcholine	−5	4	−2.25	4.6	AFDX384	−9.6	0.52	3.42	7.5
	Oxotremorine-M	−6.1	2	−1.2	5.35	AQ-RA 741	−8.1	0.52	3.53	7.4
	Pentylthio-TZTP	−7.4	6	3.68	8.6	Droxidopa	−7	0.22	−3.07	7.1
	GSK-1034702	−9.6	2	3.2	6.5	Himbacine	−7.4	0.86	4.2	6.9
	HTL9936	−9.2	7	3.94	4.7	(*S*)-Dimetindene	−9.5	0.35	3.39	6.7

The best QSAR model to determine the binding affinity (pKi) for M1-AChR consists of three descriptors [interaction energy, number of rotatable bonds (NBR), and lipophilicity (ilog*P*)] as follows:pKi = −0.31336[*E*_6ZFZ_] + 0.2092[NBR] + 0.23651[ilog*P*] + 2.5591*Q*_LOO_^2^ = 86.02; *R*_AGONIST_^2^ = 89.64; *R*_ADJ_^2^ = 88.01; *s* = 0.511; SDEC = 0.4642; *F* = 54.8 (*df* = 3.19); *DQ* = 0.012(−0.005); *R*_p_ = 0.013(0.100); *R*_*n*_ = 0.000(−0.323)

The high quadratic correlation value (*R*_AGONIST_^2^ = 89.64) indicates that the regression line fits the data perfectly. The equation has an *R*_ADJ_^2^ value of 88.01, indicating satisfactory agreement between the correlation and the variation in data. *F* = 54.8 value indicates that our model is statistically significant. *Q*_LOO_^2^ = 86.02 illustrates the stability of the model, and this value indicates that the regression model has good predictive power.

**Fig. 16:**
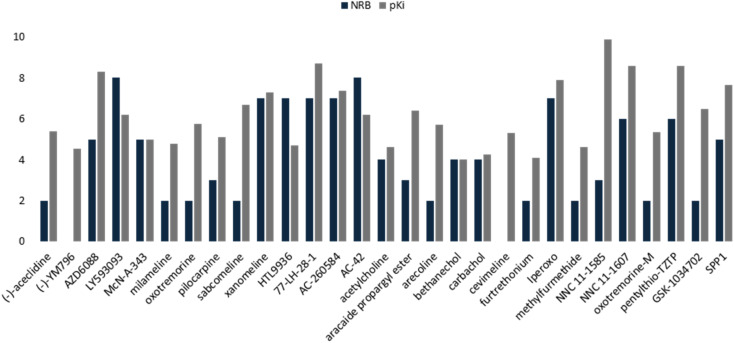
Comparative histogram of NBR and pKi values of the M1AChR agonists.

This QSAR model uses the interaction energies between the analogous molecules and M1-AChR, the more negative the interaction energy value of each molecule the higher the inhibition constant, and the better potency and affinity of the molecule. Interaction energy descriptor has a negative coefficient (−0.31336), the more negative the interaction energy the more stable the ligand-receptor complex is, therefore, and may result in an increased pKi value for the molecule. On the other hand, constitutional descriptor of number of rotatable bonds (NRB) has a positive coefficient (+0.2092); increasing NRB is detrimental to the potency. since from a thermodynamic point of view there will be a higher entropic loss if the molecule has more rotatable bonds. The increase in NRB constitutes a substantial unfavorable entropic contribution to the free energy of ligand binding. NRB is a measure of molecular flexibility and is important in determining the bioavailability of drugs.^102^ The flexibility with which a molecule crosses the BBB is related to the NRB. CNS drugs have significantly fewer spun bonds than other drug classes. More than five rotatable bonds, in centrally acting compounds, correlate with decreased facilitation with which a molecule crosses the membrane.^103^ Therefore, it does not have to be such a rigid molecule, otherwise it will not be able to adopt the right shape to interact with the receptor. In this work, a number less than or equal to five rotatable bonds is associated with higher agonist potency (Fig. 16), due to lower entropic loss of the ligand after binding. A number greater than five rotatable bonds is detrimental to agonist affinity. All compounds excluded in this model (LY593093, HTL9936, AC-42, iperoxo, NNC 11-1585, pentylthio-TZTP) have more than five rotatable bonds (Table 3).

**Fig. 17:**
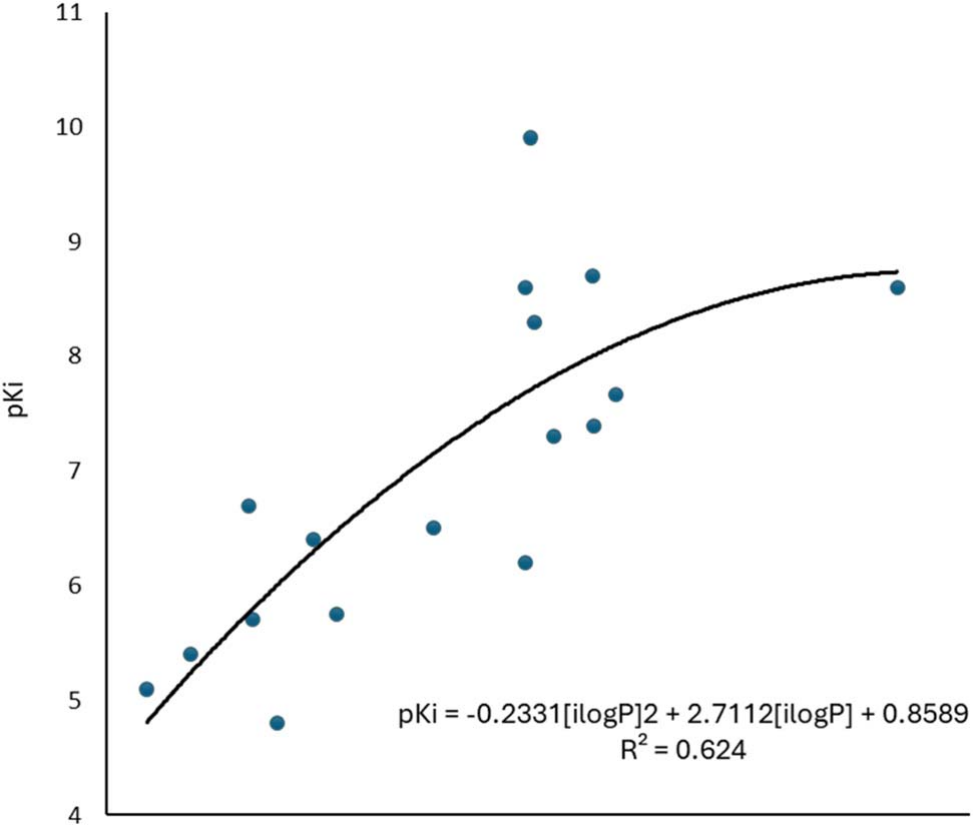
Graphical representation of the quadratic polynomial relation of the pKi of agonists to their molecular volume.

To complement the QSAR model for M1-AChR agonists, a lipophilicity descriptor, ilog*P*, was used (Table 3). The implicit log*P* method (ilog*P*) is a method that links the solvation free energy and the *n*-octanol/water partition coefficient calculated by the GB/SA [Generalized Born (GB) model augmented with the hydrophobic solvent accessible surface area (SA)] in water and *n*-octanol.^104^ The ability of drugs to cross the blood–brain barrier (BBB) is a very important pharmacokinetic indicator. Only substances that have crossed the BBB can affect physiological processes occurring in the brain.^105^ According to models previously designed to predict BBB permeability, substances with a log*P* value >0.3 cross the BBB easily, and substances with log*P* < −1.0 have difficulty crossing.^106^ Molecules with negative log*P* values have a higher preference for a hydrophilic environment (aqueous phase), whereas molecules with positive log*P* values have a higher affinity for a lipophilic environment (lipid phase). However, a very lipophilic molecule is not suitable to cross BBB. Ideally, a drug targeting the CNS should have a log*P* value of about 2.^107^ In this regard, we observed a correlation between log*P* values and affinity for M1AChR (Fig. 17).

As the molecule becomes more lipophilic, increasing the potency of the ligand. The quadratic polynomial equation of the training set (Fig. 17) shows that agonists with positive log*P* values (Table 3) have a parabolic trend suitable for obtaining the maximum value that the compounds can reach before the molecule exceeds lipophilicity and the agonist activity of the molecule fails.

**Fig. 18:**
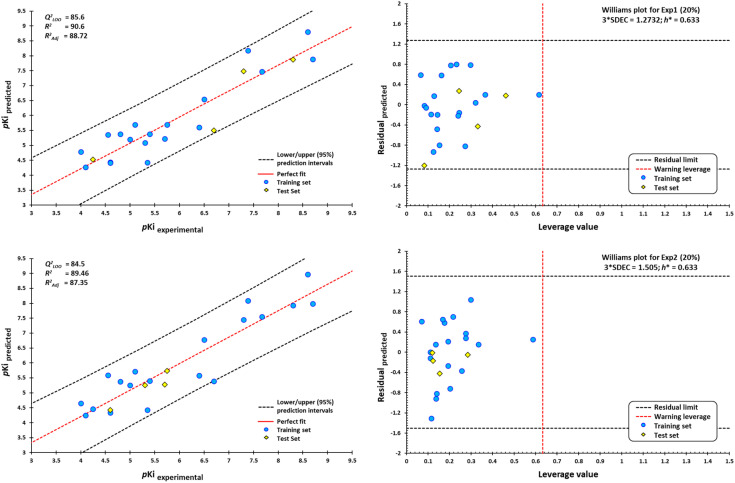
Scatterplots of predicted pKi values against experimental pKi values, and Williams plots for agonists of M1-AChR. Blue dots represent molecules of the training set (80%), and yellow diamonds depict molecules used for the test set (20%). For each plot, the percentage of molecules used in the training and test datasets were randomly chosen. The dotted vertical line in red indicates the warning leverage limit (h* = 3*p*/*n*, where *n* is the number of molecules and *p* is the number of descriptors in the model plus one). The upper/lower dotted horizontal lines in black represent the boundaries for which the triple of the standard deviation (3 × SDEC) value is used.

However, a significant increase in the value of log*P* is counterproductive for ligand affinity. NNC 11-1607 with a log*P* value = 5.62, has a receptor affinity of 8.6, whereas NNC 11-1585 with a log*P* value equal to 3.71, has a pKi = 9.9 (Table 3). Nevertheless, this approach does not apply to all removed molecules from this model, even for molecules that are within the applicability domain. Although log*P* plays a critical role in the design of new drugs, it is not the sole determinant in predicting the transport of a compound across the BBB Y-coding test was used in the training test set, giving the new values of *R*^2^ = 89.22, and *Q*_LMO_^2^ = 83.77. These new values were lower than the original values, confirming that our model is reliable. Fig. 18 shows two of at least four experiments performed for external validation.

According to the Williams plot (Fig. 18; ESI, Fig. S11†), the model does not have good prediction for sabcomeline, most likely because it is a partial agonist. Hydrophobic interactions are more frequent in highly efficient ligands.^95^ However, sabcomeline showed a limited number of hydrophobic contacts (ESI, Fig. S10†). Also, sabcomeline was unable to form pi interactions (ESI, Fig. S10†). Noncovalent interactions involving pi systems are fundamental to biological events such as protein–ligand recognition.^96^ In addition, LY593093, HTL9936, AC-42, iperoxo, NNC 11-1585, and pentylthio-TZTP fell outside the applicability domain of the QSAR model for agonists, because they presented statistical outliers to the rest of the other molecules. If the residual value of the difference between biological and predicted activity exceeds more than twice the standard deviation, can be ponder atypical molecules.^108^

Interestingly, compounds eliminated from QSAR analysis also show no correlation with the linear regression molecular docking analysis. This crosstalk in overall agonist prediction, satisfactory or not, is highly dependent on the interaction binding site volume of the co-crystallized agonists [M1R: iperoxo (PDB:6OIJ) complex, M1R: 77-LH-28-1 (PDB:6ZFZ) complex]. As described above, 77-LH-28-1, has a larger number of hydrophobic and pi interactions, which confer higher affinity and binding energy to the receptor (ESI, Fig. S10†). Most docked structures of agonists (partial or full) in M1R/iperoxo (PDB:6OIJ) complex binding site, do not have a satisfactory prediction at the structural level, nor with binding energies, due to the limited size and number of interactions generated by iperoxo, given ligand volume and binding site volume of crystal.

The shallow depth of the pockets at the receptor–ligand interface compromises their size and enclosure. Despite the structural resemblance of most experimental agonists to 77-LH-28-1 (PDB:6ZFZ), partial agonists, such as LY593093, sabcomeline, HTL9936, and full agonists, such as AC-42, iperoxo, NNC 11-1585, pentylthio-TZTP, were excluded from the QSAR model. A feature shared by all these ligands after analysis is the limited number of hydrophobic contacts (ESI, Fig. S10†). However, these are not determinative, as other factors are also involved. A functional screening analysis indicated that Y6.51 and N6.52 mutations in the orthosteric binding site of the M1-AChR did not eliminate the functional response of AC-42.^109^ Instead, mutations in the binding pocket closest to the extracellular site affected the agonism of AC-42, indicating that AC-42 binds to the site slightly above the orthosteric binding site for classical muscarinic agonists, at a site termed ectop.^109^ Subsequent optimization of the structure of AC-42 led to compound AC-260584, which binds to the same ectopic site, but with greater potency and efficacy than AC-42.^110^ In this respect, AC-42 is like 77-LH-28-1. However, unlike 77-LH-28-1, AC-42 is a biased muscarinic agonist that induces the desired outcome with limited side effects due to its different mechanism of action. Moreover, although NNC 11-1585 has a higher binding affinity (pKi = 9.9) than 77-LH-28-1 (pKi = 8.7) (Table 3), showed only one pi–alkyl interaction (Cys407), compared to NNC 11-1607 analog. The main difference between NNC 11-1585 and NNC 11-1607 is the additional presence of the quinuclidine and thiadiazole substituents in NNC 11-1607 (Fig. 1), which favors pi interactions, despite having an extremely limited number of hydrophobic contacts (ESI, Fig. S10†). Pentylthio-TZTP has similar interactions to the co-crystallized ligand, it has an unfavorable donor–donor bond with Y7.39 (Tyr404) that affects the stability of the compound's activity. All unfavorable protein–ligand interactions will reduce the stability of the complex because they indicate the repulsion that occurs between two molecules or atoms.^111^ HTL9936 is an unbiased M1 receptor partial agonist, with an alignment closer to the binding site volume of the antagonist tiotropium. The piperidine–azepine ring system of HTL9936 allows to make more contact with the major and minor pockets at the M1RAChR.^30^ Thus, HTL9936 was not related as a partial agonist for this analysis. Iperoxo had a reduced hydrophobic contact and no pi interaction (ESI, Fig. S10†). In addition, the minor pocket and extracellular vestibule are inaccessible to small molecule ligands, such as iperoxo (Table 3). The partial agonist LY593093, showed an excess of pi (pi–anion; pi–sulfur), and pi–pi (pi–pi stacked; pi–pi T-shaped) interactions, which were unfavorable for the study.

### QSAR modeling of M1-AChR antagonists

A set of 30 compounds was used in model development to evaluate the predictive ability of the QSAR model for M1AChR antagonists. The descriptors of interaction energy, fraction of sp^3^ hybridized carbons (fCsp^3^), and lipophilicity (Mlog*P*) were used in this QSAR model. The descriptors were used to generate a multilinear regression model to calculate the pKi of the M1 AChR ligands within the model chemical space. The generated model is represented by equation:pKi = −0.80323[*E*_5CXV_] + 1.6111[*f*Csp^3^] − 0.16846[Mlog*P*] + 0.71264*Q*_LOO_^2^ = 83.28; *R*_ANTAGONIST_^2^ = 88.70; *R*_ADJ_^2^ = 86.92; *s* = 0.348; FIT = 4.620; *F* = 49.7 (*df* = 3.19); *DK* = 0.0276(0.000); *R*_p_ = 0.178(0.100); *R*_*n*_ = −0.089(−0.323)

With the equation generated from the data obtained for each ligand we can infer that model is predictive. *R*_ANTAGONIST_^2^ = 88.70 value indicates that model has good descriptive power, with exceptionally good predictive power of the regression model (*Q*_LOO_^2^ = 83.28). Therefore, our work constitutes a reliable QSAR model for designing antagonists for a given target protein structure.

In this model, negative coefficient (−0.80323) of the interaction energy favors the potency, and thus the binding affinity. The bioavailability of the antagonist was assessed with the saturation descriptor, *f*Csp^3^. For saturation, the ratio of sp^3^-hybridized carbons over the total carbon count of the molecule (Csp^3^ fraction) should be no less than 0.25.^54^ Mlog*P* is the Moriguchi octanol–water partition coefficient, a measure of molecule hydrophobicity^112^ and is a frequently used descriptor in QSAR models for BBB permeability prediction.^113^ Mlog*P* suggests that only lipophilic molecules can readily penetrate BBB. Supersaturation in the molecule measured by *f*Csp^3^, is counteracted by the negative coefficient of Mlog*P* (−0.16846). An excessive increase of sp^3^-type carbon atoms in the ligand structure may impact its adsorption through the BBB.

**Fig. 19:**
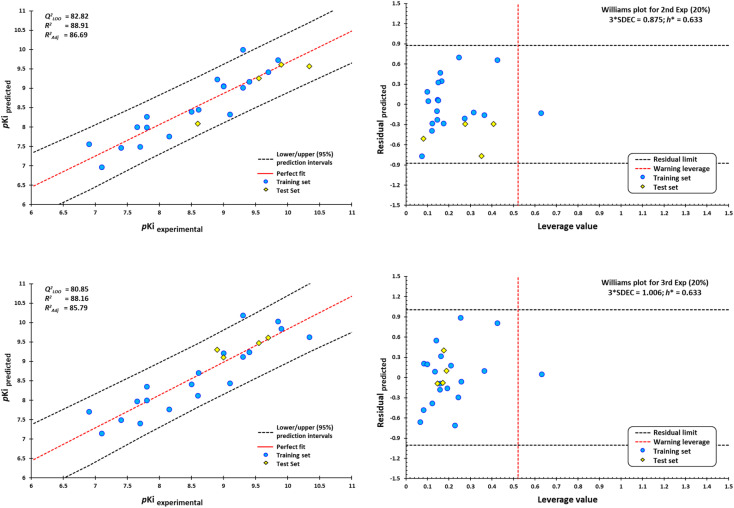
Scatterplots of predicted pKi values against experimental pKi values, and Williams plots for antagonists of M1-AChR. Blue dots represent molecules of the training set (80%), and yellow diamonds depict molecules used for the test set (20%). For each plot, the percentage of molecules used in the training and test datasets were randomly chosen. The dotted vertical line in red indicates the warning leverage limit (h* = 3*p*/*n*, where *n* is the number of molecules and *p* is the number of descriptors in the model plus one). The upper/lower dotted horizontal lines in black represent the boundaries for which the triple of the standard deviation (3 × SDEC) value is used.

External validation procedure showed upper/lower confidence intervals at the 95% confidence level (Fig. 19). The Y-coding test was used in the training test set, giving the new values of *R*^2^ = 87.7, and *Q*_LMO_^2^ = 79.26. These values indicate that our second model is reliable. The Williams plots (Fig. 19) show the composites with outliers, which the model does not predict well.

The equation was shown to be poorly predictive for 4-DAMP (ESI, Fig. S12†). This result suggests that 4-DAMP may behave as an inverse agonist. A 4-DAMP-induced reduction in receptor activity may modify binding affinities.^105,114^ However, the fact that a drug has inverse agonist properties does not mean that all responses produced by the drug are due to inverse agonism. Constitutive receptor activity is dependent on receptor density and the receptor–effector coupling efficiency; therefore, a drug with inverse agonist properties may act as an inverse agonist in some tissues and as a competitive antagonist in others, depending on the degree of constitutive receptor activity and the activity of an endogenous agonist.^115^

Seven of the 30 molecules were left out of the QSAR model because they presented outliers. Molecular docking analysis of the antagonists showed a satisfactory effect dependent on the ligand size/interaction cavity ratio. However, (*S*)-dimetindene, aclidinium, AFDX384, amitriptyline, QNB, telenzepine, and umeclidinium do not satisfy structural, binding energy, and, therefore, interaction ratio with M1AChR. The main features shared by these compounds are a deficiency of hydrophobic contacts and pi–cation interaction, and an expansion of pi–alkyl, pi–pi stacked, and pi–pi T-shaped interactions, which are unfavorable for binding at the receptor (ESI, Fig. S10†). Therefore, the model cannot be applied to the prediction of the biological activity of new compounds for the M1-AChR with high structural similarity to these seven compounds.

Furthermore, according to *in vitro* studies, it is futile to design CNS drugs by simply increasing their potency, as it results in an inconvenient increase in lipophilicity and molecular weight of the drug.^116^ One function that correlates potency, size and lipophilicity factors is the use of ligand efficiency-dependent lipophilicity, which in turn correlates potency and molecule size.^117,118^ Moreover, *in vivo* toxicological studies show a higher incidence of toxicity for lipophilic compounds.^119^ For example, the bioavailability of a drug with low aqueous solubility and high lipophilicity (logP > 3) will be alter therapeutic efficacy and drug elimination. For drugs approved in the last decade, the mean Mlog*P* value is 2.31.^120^ However, it is possible to generate drugs with some violations of the Rule of Five (Ro5) formulated by Lipinski, including low limits of lipophilicity.^121,122^ Likewise, lipophilicity is a key physicochemical parameter relating to solubility. Higher lipophilicity (log*P* > 5) correlates with lower drug solubility.^123^ The parameter *f*Csp^3^ has been related to aqueous solubility.^124,125^ The increase in saturation measured by *f*Csp^3^ and the number of chiral centers in the molecule make the compound have a rough estimate of three-dimensionality, increasing the number of possible isomers of the compound allowing enhanced interactions with the target protein, increasing the potency of the drug candidate.^126^ More than 80% of marketed drugs consider an adequate *f*Csp^3^ value ≥0.42.^127^ In this regard, the antagonists (*S*)-dimetindene, aclidinium, AFDX384, amitriptyline, QNB, telenzepine, and umeclidinium presented Mlog*P* and *f*Csp^3^ values outside the applicability of this model. (*S*)-Dimethindene, is an isomer of the histamine antagonist and has a similar ability to selectively antagonize M2 muscarinic receptors, with lower affinities for the M1 muscarinic.^128^ Of all the antagonists evaluated in this work, (*S*)-dimethindene shows lower potency for the M1-AChR (Table 3). Aclidinium bromide is a long-acting inhaled antimuscarinic agent. Although aclidinium has a comparable affinity to tiotropium (Table 3), it shows a faster onset of speed and shorter duration of action than tiotropium.^129^ However, aclidinium is rapidly hydrolyzed in human plasma to inactive metabolites.^129^ The lack of extensive hydrolysis at an adequate rate is the reason aclidinium is not involved in this QSAR model. Discussions on dosing regimens and the use of aclidinium bromide are still debated. AFDX384 has low BBB permeability and a high presence of labeled metabolites in the CNS, making it more useful in the study of muscarinic receptors present in the heart than in the brain.^130^ On the other hand, P-glycoprotein (P-gp) plays a key role in the brain transport of several antidepressants.^131^ However, the role of P-gp for amitriptyline pharmacokinetics in the CNS after chronic administration and under steady-state conditions is uncertain.^132^ It is suggested that amitriptyline induces changes in the BBB itself or that amitriptyline metabolites function as a substrate for P-gp and become oversaturated over time.^133^ In addition to amitriptyline behaving as a nonselective muscarinic antagonist, the lack of clarity regarding the status of amitriptyline as a P-gp substrate is inconsistent with the model. QNB is one of the most potent anticholinergics evaluated in this work (Table 3). It has been used as a hallucinogenic warfare agent and represents a health hazard, classified as an incapacitating agent.^134^ The neurological effects caused by QNB are due to the fact that it easily crosses the BBB, as it is highly lipophilic and has a high degree of binding to plasma proteins and red blood cells.^135^ It is a clear example that a compound with high potency is not synonymous with an effective drug. Telenzepine is an analogue of pirenzepine, and moderately reduces gastric acid secretion with no effect on blocking smooth muscle activity compared to atropine.^136^ Due to its low efficacy and undesirable anticholinergic side effects, coupled with the success of omeprazole as a more effective acid suppressant, telenzepine is no longer available for clinical use.^137^ Finally, umeclidinium was discarded from this model because it has a high molecular weight, low lipophilicity, and low solubility (*f*Csp^3^) (Table 3; ESI, Fig. S10†); moreover, its efficacy is exerted through combination with vilanterol, a β2-adrenergic agonist.^138^ In general, starting from a known antagonist, this model can be useful to improve the potency of a drug by increasing the log*P* value, without increasing the saturation levels defined by the fraction of sp^3^ hybridized carbons (*f*Csp^3^). It is important to emphasize that the molecular weight of the drug should not be increased, a key consideration for the drug-likeness noted in Ro5.

## Conclusions

In this work, our objective was to construct two QSAR models for a set of M1AChR agonists/antagonists. Descriptive models were generated with predictive ability that explain how a set of molecules has biological activity. The molecular docking results confirmed that the ligand volume is an essential factor in predicting the biological activity of the compounds studied. According to the co-crystallized ligands used in this study, there is a correlation between ligand volume and binding site volume when the binding site volume is less than or equal to 600 Å^3^. However, in none of the cases does the ligand occupy all the sites. An increase in binding site volume is detrimental to the ligand's affinity. With some exceptions, the ligand volume is related to the molecular weight (MW) of the ligand. In general, agonists and antagonists with MW between 200–400 g mol^−1^ fitted the mathematical models best, enhancing the number of pi interactions, H-bonding, and hydrophobic contacts, which contributes to the stabilization of the binding structures and enhances the ligand-receptor binding affinity. Therefore, the models presented in this work are valid and can be effectively used to predict the pKi values of new drugs.

## Author contributions

W. M.-L., R. S.-C. and R.-S. R.-H. designed the study. W. M.-L., R. S.-C., M.-I. N.-V., J.-M. A.-H. and R. S. R.-H. performed data analysis. W. M.-L. and R.-S. R.-H. wrote the manuscript. All authors revised and approved the manuscript.

## Conflicts of interest

The authors declare that there is no conflict of interest.

## Supplementary Material

RA-014-D3RA07380G-s001

## References

[cit1] Wacker D., Stevens R. C., Roth B. L. (2017). How Ligands Illuminate GPCR Molecular Pharmacology. Cell.

[cit2] Katritch V., Cherezov V., Stevens R. C. (2013). Structure-function of the G protein-coupled receptor superfamily. Annu. Rev. Pharmacol. Toxicol..

[cit3] Sriram K., Insel P. A. (2018). G Protein-Coupled Receptors as Targets for Approved Drugs: How Many Targets and How Many Drugs?. Mol. Pharmacol..

[cit4] Wess J. (2012). Novel muscarinic receptor mutant mouse models. Handb. Exp. Pharmacol..

[cit5] Wess J., Eglen R. M., Gautam D. (2007). Muscarinic acetylcholine receptors: mutant mice provide new insights for drug development. Nat. Rev. Drug Discovery.

[cit6] Langmead C. J., Watson J., Reavill C. (2008). Muscarinic acetylcholine receptors as CNS drug targets. Pharmacol. Ther..

[cit7] Ishii M., Kurachi Y. (2006). Muscarinic acetylcholine receptors. Curr. Pharm. Des..

[cit8] Hasselmo M. E. (2006). The role of acetylcholine in learning and memory. Curr. Opin. Neurobiol..

[cit9] Kruse A. C., Kobilka B. K., Gautam D., Sexton P. M., Christopoulos A., Wess J. (2014). Muscarinic acetylcholine receptors: novel opportunities for drug development. Nat. Rev. Drug Discovery.

[cit10] Warburton E. C., Koder T., Cho K., Massey P. V., Duguid G., Barker G. R. (2003). *et al.*, Cholinergic neurotransmission is essential for perirhinal cortical plasticity and recognition memory. Neuron.

[cit11] Lebois E. P., Schroeder J. P., Esparza T. J., Bridges T. M., Lindsley C. W., Conn P. J. (2017). *et al.*, Disease-Modifying Effects of M. ACS Chem. Neurosci..

[cit12] Drachman D. A., Leavitt J. (1974). Human memory and the cholinergic system. A relationship to aging?. Arch. Neurol..

[cit13] Kempuraj D., Thangavel R., Natteru P. A., Selvakumar G. P., Saeed D., Zahoor H. (2016). *et al.*, Neuroinflammation Induces Neurodegeneration. J. Neurol., Neurosurg. Psychiatry.

[cit14] Selkoe D. J., Hardy J. (2016). The amyloid hypothesis of Alzheimer's disease at 25 years. EMBO Mol. Med..

[cit15] Jack C. R., Knopman D. S., Jagust W. J., Petersen R. C., Weiner M. W., Aisen P. S. (2013). *et al.*, Tracking pathophysiological processes in Alzheimer's disease: an updated hypothetical model of dynamic biomarkers. Lancet Neurol..

[cit16] Bloom G. S. (2014). Amyloid-β and tau: the trigger and bullet in Alzheimer disease pathogenesis. JAMA Neurol.

[cit17] Knobloch M., Mansuy I. M. (2008). Dendritic spine loss and synaptic alterations in Alzheimer disease. Mol. Neurobiol..

[cit18] Yi J. H., Whitcomb D. J., Park S. J., Martinez-Perez C., Barbati S. A., Mitchell S. J. (2020). *et al.*, M1 muscarinic acetylcholine receptor dysfunction in moderate Alzheimer's disease pathology. Brain Commun..

[cit19] Felder C. C., Goldsmith P. J., Jackson K., Sanger H. E., Evans D. A., Mogg A. J. (2018). *et al.*, Current status of muscarinic M1 and M4 receptors as drug targets for neurodegenerative diseases. Neuropharmacology.

[cit20] Rook J. M., Abe M., Cho H. P., Nance K. D., Luscombe V. B., Adams J. J. (2017). *et al.*, Diverse Effects on M. ACS Chem. Neurosci..

[cit21] Moran S. P., Dickerson J. W., Cho H. P., Xiang Z., Maksymetz J., Remke D. H. (2018). et al.. Eur. Neuropsychopharmacol..

[cit22] Levey A. I. (1993). Immunological localization of m1-m5 muscarinic acetylcholine receptors in peripheral tissues and brain. Life Sci..

[cit23] Volpicelli L. A., Levey A. I. (2004). Muscarinic acetylcholine receptor subtypes in cerebral cortex and hippocampus. Prog. Brain Res..

[cit24] Konar A., Gupta R., Shukla R. K., Maloney B., Khanna V. K., Wadhwa R. (2019). *et al.*, M1 muscarinic receptor is a key target of neuroprotection, neurodegeneration and memory recovery by i-Extract from Withania somnifera. Sci. Rep..

[cit25] Zhou W., Zhu X., Zhu L., Cui Y. Y., Wang H., Qi H. (2008). *et al.*, Neuroprotection of muscarinic receptor agonist pilocarpine against glutamate-induced apoptosis in retinal neurons. Cell. Mol. Neurobiol..

[cit26] Bradley S. J., Bourgognon J. M., Sanger H. E., Verity N., Mogg A. J., White D. J. (2017). *et al.*, M1 muscarinic allosteric modulators slow prion neurodegeneration and restore memory loss. J. Clin. Invest..

[cit27] Fisher A., Bezprozvanny I., Wu L., Ryskamp D. A., Bar-Ner N., Natan N. (2016). *et al.*, AF710B, a Novel M1/σ1 Agonist with Therapeutic Efficacy in Animal Models of Alzheimer's Disease. Neurodegener. Dis..

[cit28] Shirey J. K., Brady A. E., Jones P. J., Davis A. A., Bridges T. M., Kennedy J. P. (2009). *et al.*, A selective allosteric potentiator of the M1 muscarinic acetylcholine receptor increases activity of medial prefrontal cortical neurons and restores impairments in reversal learning. J. Neurosci..

[cit29] Dwomoh L., Rossi M., Scarpa M., Khajehali E., Molloy C., Herzyk P. (2022). et al.. Sci. Signaling.

[cit30] Brown A. J. H., Bradley S. J., Marshall F. H., Brown G. A., Bennett K. A., Brown J. (2021). *et al.*, From structure to clinic: Design of a muscarinic M1 receptor agonist with potential to treatment of Alzheimer's disease. Cell.

[cit31] Thal D. M., Sun B., Feng D., Nawaratne V., Leach K., Felder C. C. (2016). *et al.*, Crystal structures of the M1 and M4 muscarinic acetylcholine receptors. Nature.

[cit32] Haga K., Kruse A. C., Asada H., Yurugi-Kobayashi T., Shiroishi M., Zhang C. (2012). *et al.*, Structure of the human M2 muscarinic acetylcholine receptor bound to an antagonist. Nature.

[cit33] Kruse A. C., Hu J., Pan A. C., Arlow D. H., Rosenbaum D. M., Rosemond E. (2012). *et al.*, Structure and dynamics of the M3 muscarinic acetylcholine receptor. Nature.

[cit34] Hulme E. C., Birdsall N. J., Buckley N. J. (1990). Muscarinic receptor subtypes. Annu. Rev. Pharmacol. Toxicol..

[cit35] Hulme E. C., Lu Z. L., Saldanha J. W., Bee M. S. (2003). Structure and activation of muscarinic acetylcholine receptors. Biochem. Soc. Trans..

[cit36] Chintamaneni P. K., Krishnamurthy P. T., Rao P. V., Pindiprolu S. S. (2017). Surface-modified nano-lipid drug conjugates of positive allosteric modulators of M1 muscarinic acetylcholine receptor for the treatment of Alzheimer's disease. Med. Hypotheses.

[cit37] Urniaż R. D., Jóźwiak K. (2013). X-ray crystallographic structures as a source of ligand alignment in 3D-QSAR. J. Chem. Inf. Model..

[cit38] Tropsha A., Golbraikh A. (2007). Predictive QSAR modeling workflow, model applicability domains, and virtual screening. Curr. Pharm. Des..

[cit39] Martínez-Campos Z., Pastor N., Pineda-Urbina K., Gómez-Sandoval Z., Fernández-Zertuche M., Razo-Hernández R. S. (2019). In silico structure-based design of GABA. Chem. Biol. Drug Des..

[cit40] Marbán-González A., Hernández-Mendoza A., Ordóñez M., Razo-Hernández R. S., Viveros-Ceballos J. L. (2021). Discovery of Octahydroisoindolone as a Scaffold for the Selective Inhibition of Chitinase B1 from. Molecules.

[cit41] Nolasco-Quintana N. Y., González-Maya L., Razo-Hernández R. S., Alvarez L. (2023). Exploring the Gallic and Cinnamic Acids Chimeric Derivatives as Anticancer Agents over HeLa Cell Line: An *in silico* and *in vitro* Study. Mol. Inf..

[cit42] Morris G. M., Lim-Wilby M. (2008). Molecular docking. Methods Mol. Biol..

[cit43] Meng X. Y., Zhang H. X., Mezei M., Cui M. (2011). Molecular docking: a powerful approach for structure-based drug discovery. Curr. Comput.-Aided Drug Des..

[cit44] Gaulton A., Hersey A., Nowotka M., Bento A. P., Chambers J., Mendez D. (2017). *et al.*, The ChEMBL database in 2017. Nucleic Acids Res..

[cit45] Rüdiger Bauernschmitt R. A. (1996). Treatment of electronic excitations within the adiabatic approximation of time dependent density functional theory. Chem. Phys. Lett..

[cit46] Hernández-López H., Leyva-Ramos S., Azael Gómez-Durán C. F., Pedraza-Alvarez A., Rodríguez-Gutiérrez I. R., Leyva-Peralta M. A. (2020). *et al.*, Synthesis of 1,4-Biphenyl-triazole Derivatives as Possible 17β-HSD1 Inhibitors: An. ACS Omega.

[cit47] Berman H. M., Westbrook J., Feng Z., Gilliland G., Bhat T. N., Weissig H. (2000). *et al.*, The Protein Data Bank. Nucleic Acids Res..

[cit48] Berman H., Henrick K., Nakamura H. (2003). Announcing the worldwide Protein Data Bank. Nat. Struct. Biol..

[cit49] Brown A. J. H., Bradley S. J., Marshall F. H., Brown G. A., Bennett K. A., Brown J. (2021). *et al.*, From structure to clinic: Design of a muscarinic M1 receptor agonist with potential to treatment of Alzheimer's disease. Cell.

[cit50] Maeda S., Xu J., N Kadji F. M., Clark M. J., Zhao J., Tsutsumi N. (2020). *et al.*, Structure and selectivity engineering of the M. Science.

[cit51] Maeda S., Qu Q., Robertson M. J., Skiniotis G., Kobilka B. K. (2019). Structures of the M1 and M2muscarinic acetylcholine receptor/G-protein complexes. Science.

[cit52] Trott O., Olson A. J. (2010). AutoDock Vina: improving the speed and accuracy of docking with a new scoring function, efficient optimization, and multithreading. J. Comput. Chem..

[cit53] BIOVIA , Dassault Systèmes. Discovery Studio Visualizer, v21.1.0.20298, Dassault Systèmes, San Diego, CA, USA, 2021, https://discover.3ds.com/discovery-studio-visualizer-download, accessed on 21 December 2021

[cit54] Daina A., Michielin O., Zoete V. (2017). SwissADME: a free web tool to evaluatepharmacokinetics, drug-likeness and medicinal chemistry friendliness of small molecules. Sci. Rep..

[cit55] Dong J., Cao D. S., Miao H. Y., Liu S., Deng B. C., Yun Y. H. (2015). *et al.*, ChemDes: an integrated web-based platform for molecular descriptor and fingerprint computation. J. Cheminf..

[cit56] Palacios-Can F. J., Silva-Sánchez J., León-Rivera I., Tlahuext H., Pastor N., Razo-Hernández R. S. (2023). Identification of a Family of Glycoside Derivatives Biologically Active against. Pharmaceuticals.

[cit57] Ventura-Salazar I. A. Y., Palacios-Can F. J., González-Maya L., Sánchez-Carranza J. N., Antunez-Mojica M., Razo-Hernández R. S. (2023). *et al.*, Finding a Novel Chalcone-Cinnamic Acid Chimeric Compound with Antiproliferative Activity against MCF-7 Cell Line Using a Free-Wilson Type Approach. Molecules.

[cit58] Vass M., Podlewska S., de Esch I. J. P., Bojarski A. J., Leurs R., Kooistra A. J. (2019). *et al.*, Aminergic GPCR-Ligand Interactions: A Chemical and Structural Map of Receptor Mutation Data. J. Med. Chem..

[cit59] Ballesteros Juan A. W. H. , Integrated methods for the construction of three-dimensional models and computational probing of structure-function relations in G protein-coupled receptors, in Methods in Neurosciences, ed. S. C. Sealfon, Academic Press, 1995, vol. 25, pp. 366–428

[cit60] Wess J., Gdula D., Brann M. R. (1991). Site-directed mutagenesis of the m3 muscarinic receptor: identification of a series of threonine and tyrosine residues involved in agonist but not antagonist binding. EMBO J..

[cit61] Heitz F., Holzwarth J. A., Gies J. P., Pruss R. M., Trumpp-Kallmeyer S., Hibert M. F. (1999). *et al.*, Site-directed mutagenesis of the putative human muscarinic M2 receptor binding site. Eur. J. Pharmacol..

[cit62] Venkatakrishnan A. J., Deupi X., Lebon G., Tate C. G., Schertler G. F., Babu M. M. (2013). Molecular signatures of G-protein-coupled receptors. Nature.

[cit63] Schwartz T. W., Frimurer T. M., Holst B., Rosenkilde M. M., Elling C. E. (2006). Molecular mechanism of 7TM receptor activation – a global toggle switch model. Annu. Rev. Pharmacol. Toxicol..

[cit64] Wess J., Nanavati S., Vogel Z., Maggio R. (1993). Functional role of proline and tryptophan residues highly conserved among G protein-coupled receptors studied by mutational analysis of the m3 muscarinic receptor. EMBO J..

[cit65] Goodwin J. A., Hulme E. C., Langmead C. J., Tehan B. G. (2007). Roof and floor of the muscarinic binding pocket: variations in the binding modes of orthosteric ligands. Mol. Pharmacol..

[cit66] Tautermann C. S., Kiechle T., Seeliger D., Diehl S., Wex E., Banholzer R. (2013). *et al.*, Molecular basis for the long duration of action and kinetic selectivity of tiotropium for the muscarinic M3 receptor. J. Med. Chem..

[cit67] Ward S. D., Curtis C. A., Hulme E. C. (1999). Alanine-scanning mutagenesis of transmembrane domain 6 of the M(1) muscarinic acetylcholine receptor suggests that Tyr381 plays key roles in receptor function. Mol. Pharmacol..

[cit68] Vogel W. K., Sheehan D. M., Schimerlik M. I. (1997). Site-directed mutagenesis on the m2 muscarinic acetylcholine receptor: the significance of Tyr403 in the binding of agonists and functional coupling. Mol. Pharmacol..

[cit69] Bourdon H., Trumpp-Kallmeyer S., Schreuder H., Hoflack J., Hibert M., Wermuth C. G. (1997). Modelling of the binding site of the
human m1 muscarinic receptor: experimental validation and refinement. J. Comput.-Aided Mol. Des..

[cit70] Huang X. P., Nagy P. I., Williams F. E., Peseckis S. M., Messer W. S. (1999). Roles of threonine 192 and asparagine 382 in agonist and antagonist interactions with M1 muscarinic receptors. Br. J. Pharmacol..

[cit71] Messerer R., Kauk M., Volpato D., Alonso Canizal M. C., Klöckner J., Zabel U. (2017). *et al.*, FRET Studies of Quinolone-Based Bitopic Ligands and Their Structural Analogues at the Muscarinic M. ACS Chem. Biol..

[cit72] Chen X., Klöckner J., Holze J., Zimmermann C., Seemann W. K., Schrage R. (2015). *et al.*, Rational design of partial agonists for the muscarinic m1 acetylcholine receptor. J. Med. Chem..

[cit73] Bock A., Merten N., Schrage R., Dallanoce C., Bätz J., Klöckner J. (2012). *et al.*, The allosteric vestibule of a seven-transmembrane helical receptor controls G-protein coupling. Nat. Commun..

[cit74] Schrage R., Seemann W. K., Klöckner J., Dallanoce C., Racké K., Kostenis E. (2013). *et al.*, Agonists with supraphysiological efficacy at the muscarinic M2 ACh receptor. Br. J. Pharmacol..

[cit75] Kruse A. C., Ring A. M., Manglik A., Hu J., Hu K., Eitel K. (2013). *et al.*, Activation and allosteric modulation of a muscarinic acetylcholine receptor. Nature.

[cit76] Lebon G., Langmead C. J., Tehan B. G., Hulme E. C. (2009). Mutagenic mapping suggests a novel binding mode for selective agonists of M1 muscarinic acetylcholine receptors. Mol. Pharmacol..

[cit77] Hollingsworth S. A., Kelly B., Valant C., Michaelis J. A., Mastromihalis O., Thompson G. (2019). *et al.*, Cryptic pocket formation underlies allosteric modulator selectivity at muscarinic GPCRs. Nat. Commun..

[cit78] Abdul-Ridha A., López L., Keov P., Thal D. M., Mistry S. N., Sexton P. M. (2014). *et al.*, Molecular determinants of allosteric modulation at the M1 muscarinic acetylcholine receptor. J. Biol. Chem..

[cit79] Bee M. S., Hulme E. C. (2007). Functional analysis of transmembrane domain 2 of the M1 muscarinic acetylcholine receptor. J. Biol. Chem..

[cit80] Schmitz J., van der Mey D., Bermudez M., Klöckner J., Schrage R., Kostenis E. (2014). *et al.*, Dualsteric muscarinic antagonists-orthosteric binding pose controls allosteric subtype selectivity. J. Med. Chem..

[cit81] Keov P., López L., Devine S. M., Valant C., Lane J. R., Scammells P. J. (2014). *et al.*, Molecular mechanisms of bitopic ligand engagement with the M1 muscarinic acetylcholine receptor. J. Biol. Chem..

[cit82] Isberg V., de Graaf C., Bortolato A., Cherezov V., Katritch V., Marshall F. H. (2015). *et al.*, Generic GPCR residue numbers – aligning topology maps while minding the gaps. Trends Pharmacol. Sci..

[cit83] Leach K., Davey A. E., Felder C. C., Sexton P. M., Christopoulos A. (2011). The role of transmembrane domain 3 in the actions of orthosteric, allosteric, and atypical agonists of the M4 muscarinic acetylcholine receptor. Mol. Pharmacol..

[cit84] Hulme E. C., Curtis C. A., Page K. M., Jones P. G. (1995). The role of charge interactions in muscarinic agonist binding, and receptor-response coupling. Life Sci..

[cit85] Canal C. E., Cordova-Sintjago T. C., Villa N. Y., Fang L. J., Booth R. G. (2011). Drug discovery targeting human 5-HT(2C) receptors: residues S3.36 and Y7.43 impact ligand-binding pocket structure *via* hydrogen bond formation. Eur. J. Pharmacol..

[cit86] De Amici M., Dallanoce C., Holzgrabe U., Tränkle C., Mohr K. (2010). Allosteric ligands for G protein-coupled receptors: a novel strategy with attractive therapeutic opportunities. Med. Res. Rev..

[cit87] Digby G. J., Shirey J. K., Conn P. J. (2010). Allosteric activators of muscarinic receptors as novel approaches for treatment of CNS disorders. Mol. BioSyst..

[cit88] Gregory K. J., Sexton P. M., Christopoulos A. (2007). Allosteric modulation of muscarinic acetylcholine receptors. Curr. Neuropharmacol..

[cit89] Wheatley M., Wootten D., Conner M. T., Simms J., Kendrick R., Logan R. T. (2012). *et al.*, Lifting the lid on GPCRs: the role of extracellular loops. Br. J. Pharmacol..

[cit90] Kappel K., Miao Y., McCammon J. A. (2015). Accelerated molecular dynamics simulations of ligand binding to a muscarinic G-protein-coupled receptor. Q. Rev. Biophys..

[cit91] Blüml K., Mutschler E., Wess J. (1994). Functional role in ligand binding and receptor activation of an asparagine residue present in the sixth transmembrane domain of all muscarinic acetylcholine receptors. J. Biol. Chem..

[cit92] Kitchen D. B., Decornez H., Furr J. R., Bajorath J. (2004). Docking and scoring in virtual screening for drug discovery: methods and applications. Nat. Rev. Drug Discovery.

[cit93] Carter-Fenk K., Herbert J. M. (2020). Electrostatics does not dictate the slip-stacked arrangement of aromatic π-π interactions. Chem. Sci..

[cit94] Brylinski M. (2018). Aromatic interactions at the ligand-protein interface: implications for the development of docking scoring functions. Chem. Biol. Drug Des..

[cit95] Ferreira de Freitas R., Schapira M. (2017). A systematic analysis of atomic protein-ligand interactions in the PDB. MedChemComm.

[cit96] Yang D., Zhou Q., Labroska V., Qin S., Darbalaei S., Wu Y. (2021). *et al.*, G protein-coupled receptors: structure- and function-based drug discovery. Signal Transduction Targeted Ther..

[cit97] Infield D. T., Rasouli A., Galles G. D., Chipot C., Tajkhorshid E., Ahern C. A. (2021). Cation-π Interactions and their Functional Roles in Membrane Proteins. J. Mol. Biol..

[cit98] Wang H., Wang W., Jin W. J. (2016). σ-Hole Bond *vs.* π-Hole Bond: A Comparison Based on Halogen Bond. Chem. Rev..

[cit99] Ringer A. L., Figgs M. S., Sinnokrot M. O., Sherrill C. D. (2006). Aliphatic C-H/pi interactions: methane-benzene, methane-phenol, and methane-indole complexes. J. Phys. Chem. A.

[cit100] Viguera A. R., Serrano L. (1995). Side-chain interactions between sulfur-containing aminoacids and phenylalanine in alpha-helices. Biochemistry.

[cit101] Zauhar R. J., Colbert C. L., Morgan R. S., Welsh W. J. (2000). Evidence for a strong sulfur-aromatic interaction derived from crystallographic data. Biopolymers.

[cit102] Veber D. F., Johnson S. R., Cheng H. Y., Smith B. R., Ward K. W., Kopple K. D. (2002). Molecular properties that influence the oral bioavailability of drug candidates. J. Med. Chem..

[cit103] Leeson P. D., Davis A. M. (2004). Time-related differences in the physical property profiles of oral drugs. J. Med. Chem..

[cit104] Daina A., Michielin O., Zoete V. (2014). iLOGP: a simple, robust, and efficient description of n-octanol/water partition coefficient for drug design using the GB/SA approach. J. Chem. Inf. Model..

[cit105] Bagchi S., Chhibber T., Lahooti B., Verma A., Borse V., Jayant R. D. (2019). *In vitro* blood–brain barrier models for drug screening and permeation studies: an overview. Drug Des., Dev. Ther..

[cit106] Vilar S., Chakrabarti M., Costanzi S. (2010). Prediction of passive blood–brain partitioning: straightforward and effective classification models based on *in silico* derived physicochemical descriptors. J. Mol. Graphics Modell..

[cit107] Hansch C., Steward A. R., Anderson S. M., Bentley D. (1968). The parabolic dependence of drug action upon lipophilic character as revealed by a study of hypnotics. J. Med. Chem..

[cit108] Sarkar A., Middya T. R., Jana A. D. (2012). A QSAR study of radical scavenging antioxidant activity of a series of flavonoids using DFT based quantum chemical descriptors-the importance of group frontier electron density. J. Mol. Model..

[cit109] Spalding T. A., Trotter C., Skjaerbaek N., Messier T. L., Currier E. A., Burstein E. S. (2002). *et al.*, Discovery of an ectopic activation site on the M(1) muscarinic receptor. Mol. Pharmacol..

[cit110] Spalding T. A., Ma J. N., Ott T. R., Friberg M., Bajpai A., Bradley S. R. (2006). *et al.*, Structural requirements of transmembrane domain 3 for activation by the M1 muscarinic receptor agonists AC-42, AC-260584, clozapine, and N-desmethylclozapine: evidence for three distinct modes of receptor activation. Mol. Pharmacol..

[cit111] Halder S. T., Dhorajiwala T. M., Samant L. R. (2019). Multiple docking analysis and. Int. J. Mycobact..

[cit112] Tshepelevitsh S., Kadam S. A., Darnell A., Bobacka J., Rüütel A., Haljasorg T. (2020). *et al.*, LogP determination for highly lipophilic hydrogen-bonding anion receptor molecules. Anal. Chim. Acta.

[cit113] Zhang L., Zhu H., Oprea T. I., Golbraikh A., Tropsha A. (2008). QSAR modeling of the blood–brain barrier permeability for diverse organic compounds. Pharm. Res..

[cit114] Dowling M. R., Willets J. M., Budd D. C., Charlton S. J., Nahorski S. R., Challiss R. A. (2006). A single point mutation (N514Y) in the human M3 muscarinic acetylcholine receptor reveals differences in the properties of antagonists: evidence for differential inverse agonism. J. Pharmacol. Exp. Ther..

[cit115] Berg K. A., Clarke W. P. (2018). Making Sense of Pharmacology: Inverse Agonism and Functional Selectivity. Int. J. Neuropsychopharmacol..

[cit116] Abad-Zapatero C. (2007). Ligand efficiency indices for effective drug discovery. Expert Opin. Drug Discovery.

[cit117] Keserü G. M., Makara G. M. (2009). The influence of lead discovery strategies on the properties of drug candidates. Nat. Rev. Drug Discovery.

[cit118] Hopkins A. L., Keserü G. M., Leeson P. D., Rees D. C., Reynolds C. H. (2014). The role of ligand efficiency metrics in drug discovery. Nat. Rev. Drug Discovery.

[cit119] Hughes J. D., Blagg J., Price D. A., Bailey S., Decrescenzo G. A., Devraj R. V. (2008). *et al.*, Physiochemical drug properties associated with *in vivo* toxicological
outcomes. Bioorg. Med. Chem. Lett..

[cit120] Tsantili-Kakoulidou A., Demopoulos V. J. (2021). Drug-like Properties and Fraction Lipophilicity Index as a combined metric. ADMET DMPK.

[cit121] Young R. J., Green D. V., Luscombe C. N., Hill A. P. (2011). Getting physical in drug discovery II: the impact of chromatographic hydrophobicity measurements and aromaticity. Drug Discovery Today.

[cit122] Motoyama K., Nagata T., Kobayashi J., Nakamura A., Miyoshi N., Kazui M. (2018). *et al.*, Discovery of a bicyclo[4.3.0]nonane derivative DS88790512 as a potent, selective, and orally bioavailable blocker of transient receptor potential canonical 6 (TRPC6). Bioorg. Med. Chem. Lett..

[cit123] Remko M., Swart M., Bickelhaupt F. M. (2006). Theoretical study of structure, pKa, lipophilicity, solubility, absorption, and polar surface area of some centrally acting antihypertensives. Bioorg. Med. Chem..

[cit124] Lovering F., Bikker J., Humblet C. (2009). Escape from flatland: increasing saturation as anapproach to improving clinical success. J. Med. Chem..

[cit125] Yan A., Gasteiger J., Krug M., Anzali S. (2004). Linear and nonlinear functions on modeling of aqueous solubility of organic compounds by two structure representation methods. J. Comput.-Aided Mol. Des..

[cit126] Waring M. J., Arrowsmith J., Leach A. R., Leeson P. D., Mandrell S., Owen R. M. (2015). *et al.*, An analysis of the attrition of drug candidates from four major pharmaceutical companies. Nat. Rev. Drug Discovery.

[cit127] Shultz M. D. (2019). Two Decades under the Influence of the Rule of Five and the Changing Properties of Approved Oral Drugs. J. Med. Chem..

[cit128] Böhme T. M., Keim C., Kreutzmann K., Linder M., Dingermann T., Dannhardt G. (2003). *et al.*, Structure-activity relationships of dimethindene derivatives as new M2-selective muscarinic receptor antagonists. J. Med. Chem..

[cit129] Gavaldà A., Ramos I., Carcasona C., Calama E., Otal R., Montero J. L. (2014). *et al.*, The *in vitro* and *in vivo* profile of aclidinium bromide in comparison with glycopyrronium bromide. Pulm. Pharmacol. Ther..

[cit130] Mickala P., Boutin H., Bellanger C., Chevalier C., MacKenzie E. T., Dauphin F. (1996). In vivo binding, pharmacokinetics and metabolism of the selective M2 muscarinic antagonists[3H]AF-DX 116 and [3H]AF-DX 384 in the anesthetized rat. Nucl. Med. Biol..

[cit131] O'Brien F. E., Dinan T. G., Griffin B. T., Cryan J. F. (2012). Interactions between antidepressants and P-glycoprotein at the blood–brain barrier: clinical significance of *in vitro* and *in vivo* findings. Br. J. Pharmacol..

[cit132] Uhr M., Grauer M. T., Yassouridis A., Ebinger M. (2007). Blood–brain barrier penetration and pharmacokinetics of amitriptyline and its metabolites in p-glycoprotein (abcb1ab) knock-out mice and controls. J. Psychiatr. Res..

[cit133] Uhr M., Steckler T., Yassouridis A., Holsboer F. (2000). Penetration of amitriptyline, but not of fluoxetine, into brain is enhanced in mice with blood–brain barrier deficiency due to mdr1aP-glycoprotein gene disruption. Neuropsychopharmacology.

[cit134] Uher M., Mžik M., Karasová J., Herman D., Čechová L., Dlabková A. (2020). *et al.*, *In vitro* and *in vivo* metabolism of 3-quinuclidinyl benzilate by high-resolution mass spectrometry. J. Pharm. Biomed. Anal..

[cit135] Jing Liu L. K. W. and Pope C. N., Chemical warfare agents and the nervous system, in Handbook of Toxicology of Chemical Warfare Agents, ed. R. C. Gupta, Academic Press, 3rd edn, 2020, pp. 481–98

[cit136] Stockbrügger R. W. (1988). Clinical significance of M1 receptor antagonists. Pharmacology.

[cit137] Busam J., Garbe W. E. (1994). [High-dose omeprazole *versus* famotidine, pirenzepine and antacid in therapy of acute upper gastrointestinal hemorrhage in a retrospective comparison]. Z. Gastroenterol..

[cit138] Feldman G. J., Edin A. (2013). The combination of umeclidinium bromide and vilanterol in the management of chronic obstructive pulmonary disease: current evidence and future prospects. Ther. Adv. Respir. Dis..

