# Supplementary material for: Analysing the effect caused by increasing the molecular volume in M1-AChR receptor agonists and antagonists: a structural and computational study
Source: RSC Adv. 2024 Mar 14;14(13):8615–40. doi: 10.1039/d3ra07380g (PMC10938299; doi:10.1039/d3ra07380g)

Electronic Supplementary Material (ESI) for RSC Advances.
This journal is © The Royal Society of Chemistry 2024

Analysing the effect caused by increasing the molecular volume in M1-AChR receptor agonists and antagonists: A structural and computational study.

Supplementary information

Figure-S1 Potential binding pockets in the muscarinic receptor.

PDB	Subtype M receptor	Ligand	Drug score	Volume [Å³]	Surface [Å²]	Hydrophobicity ratio
6OIJ	M1R	Iperoxo	0.57	481.44	416.59	0.26
6ZFZ	M1R	77-LH-28-1	0.71	853.36	846.79	0.34
6WJC	M1R	Atropine	0.81	1271.94	1097.9	0.33
5CXV	M1R	Tiotropium	0.56	693.47	568.59	0.29
4MQS	M2R	Iperoxo	0.54	391.02	274.65	0.17

Figure-S2 General data of agonists/antagonists at the human M1 muscarinic receptor. Δv, volume difference to the co-crystallized ligand, and experimental data of binding energies (kcal/mol) of selected molecule ligands known to target M1-AChR discovered in the Docking Screens.

	**Agonist**						**Binding energy (kcal/mol)**	
	**Molecule**	**pKi**	**MW (g/mol)**	**Volume [Å³]**	**Δv 6OIJ Iperoxo**	**Δv 6ZFZ 77-LH-28-1**	**6OIJ**	**6ZFZ**
Partial agonist	AZD6088	8.3	393.55	416.01	-195.31	-36.51	-7.1	-11.3
	SPP1	7.67	427.57	454.12	-233.42	-74.62	-7.7	-9.6
	xanomeline	7.3	282.43	299.97	-79.27	79.53	-5.8	-8
	sabcomeline	6.7	194.26	207.12	13.58	172.38	-4.9	-6.7
	LY593093	6.2	508.62	534.58	-313.88	-155.08	-8.5	-11.8
	oxotremorine	5.75	207.30	235.81	-15.11	143.69	-8	-7
	(-)-aceclidine	5.4	170.23	181.6	39.10	197.90	-5.4	-6.5
	pilocarpine	5.1	209.27	222.06	-1.36	157.44	-6.2	-6.8
	McN-A-343	5	281.76	291.67	-70.97	87.83	-6.9	-8
	milameline	4.8	155.22	178.32	42.38	201.18	-5.9	-5.8
	HTL9936	4.7	366.53	400.35	-179.65	-20.85	-7.2	-9.2
	(-)-YM796	4.55	182.29	213.06	7.64	166.44	-4.1	-6.9
Full agonist	NNC 11-1585	9.9	326.44	347.76	-127.06	31.74	-7.3	-9.6
	77-LH-28-1	8.7	329.51	379.5	-158.80	0.00	-5	-10.1
	NNC 11-1607	8.6	574.77	564.73	-344.03	-185.23	-8.5	-11.9
	pentylthio-TZTP	8.6	284.47	292.15	-71.45	87.35	-5.6	-7.4
	Iperoxo	7.89	197.26	220.7	0.00	158.80	-6.1	-5.9
	AC-260584	7.39	348.46	375.46	-154.76	4.04	-5.8	-9.5
	GSK-1034702	6.5	334.42	339.04	-118.34	40.46	-6.1	-9.6
	aracaide propargyl ester	6.4	180.23	202.15	18.55	177.35	-6.6	-6.3
	AC-42	6.2	302.48	359.37	-138.67	20.13	-7.1	-9.4
	arecoline	5.7	156.21	172.67	48.03	206.83	-5.5	-5.9
	oxotremorine-M	5.35	195.29	229.41	-8.71	150.09	-2.5	-7
	cevimeline	5.3	200.33	204.46	16.24	175.04	-6.7	-6.1
	HTL9936	4.7	366.53	400.35	-179.65	-20.85	-7.2	-9.2
	acetylcholine	4.6	146.21	171.6	49.10	207.90	-4.5	-5
	methylfurmethide	4.6	154.23	185.82	34.88	193.68	-4.9	-5.7
	carbachol	4.25	147.20	163.41	57.29	216.09	-4.4	-4.9
	furtrethonium	4.1	140.21	167.71	52.99	211.79	-4.2	-5.3
	bethanechol	4	161.23	181.39	39.31	198.11	-4.3	-5.4

**Antagonist**						**Binding energy (kcal/mol)**	
**Molecule**	**pKi**	**MW (g/mol)**	**Volume [Å³]**	**Δv 6WJC Atropine**	**Δv 5CXV Tiotropium**	**6WJC**	**5CXV**
QNB	10.7	338.43	358.40	-49.25	36.72	-10.2	-10.7
tiotropium	10.34	392.52	395.12	-85.97	0.00	-8.8	-9.8
aclidinium	10.2	484.66	533.22	-224.07	-138.10	-7.7	-8
N-methyl scopolamine	9.9	318.39	330.56	-21.41	64.56	-8.6	-9.7
glycopyrrolate	9.85	318.44	349.19	-40.04	45.93	-8.8	-10.1
umeclidinium	9.8	428.60	475.09	-165.94	-79.97	-9.6	-9.4
propantheline	9.7	368.50	407.36	-98.21	-12.24	-9.6	-10.2
ipratropium	9.55	332.46	365.56	-56.41	29.56	-9	-9.4
revefenacin	9.4	599.78	636.06	-326.91	-240.94	-8.6	-10.4
Telenzepine	9.4	372.49	372.03	-62.88	23.09	-8.1	-7.8
4-DAMP	9.3	324.44	362.79	-53.64	32.33	-9.5	-10.8
biperiden	9.3	312.48	354.08	-44.93	41.04	-9.4	-10
atropine	9.1	290.38	309.15	0.00	85.97	-8.3	-8.9
benzatropine	9	308.45	348.43	-39.28	46.69	-9.7	-10.3
scopolamine	9	304.37	312.32	-3.17	82.80	-8.5	-9.6
trihexyphenidyl	8.9	302.48	350.58	-41.43	44.54	-9.3	-10
dicyclomine	8.61	310.50	363.83	-54.68	31.29	-8.6	-8.7
tolterodine	8.5	358.50	387.97	-78.82	7.15	-8.7	-9.6
oxybutynin	8.45	326.50	406.22	-97.07	-11.10	-8.5	-8.7
pirenzepine	8.15	353.43	360.26	-51.11	34.86	-8.5	-8.6
amitriptyline	7.8	278.42	327.15	-18.00	67.97	-10.1	-10.3
solifenacin	7.8	363.48	387.35	-78.20	7.77	-8.4	-8.9
VU0255035	7.8	433.54	394.36	-85.21	0.76	-8.9	-8.7
dosulepin	7.7	296.46	328.22	-19.07	66.90	-9.2	-8.8
darifenacin	7.65	427.57	460.81	-151.66	-65.69	-9.9	-9.1
AFDX384	7.5	480.66	517.87	-208.72	-122.75	-9	-9.6
AQ-RA 741	7.4	464.63	502.39	-193.24	-107.27	-8.1	-8.1
droxidopa	7.1	213.19	195.45	113.70	199.67	-6.8	-7
himbacine	6.9	348.55	393.32	-84.17	1.80	-7.4	-7.4
(S)-dimetindene	6.7	293.43	340.13	-30.98	54.99	-8.8	-9.5

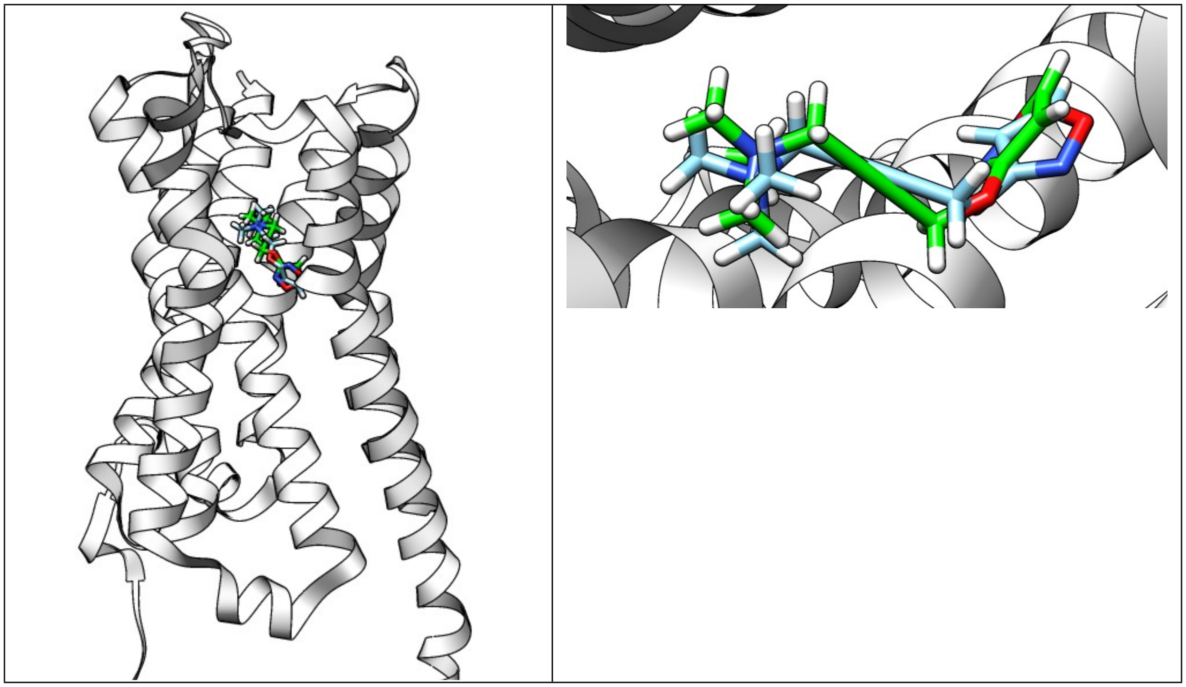

Figure-S3 Crystal structure of the M1AChR-iperoxo complex (PDB:6OIJ) with the overlapping ligand pose obtained from docking (RMSD=0.82 Å). The conformation obtained from the docking calculation is green, and the crystal conformation is cyan.

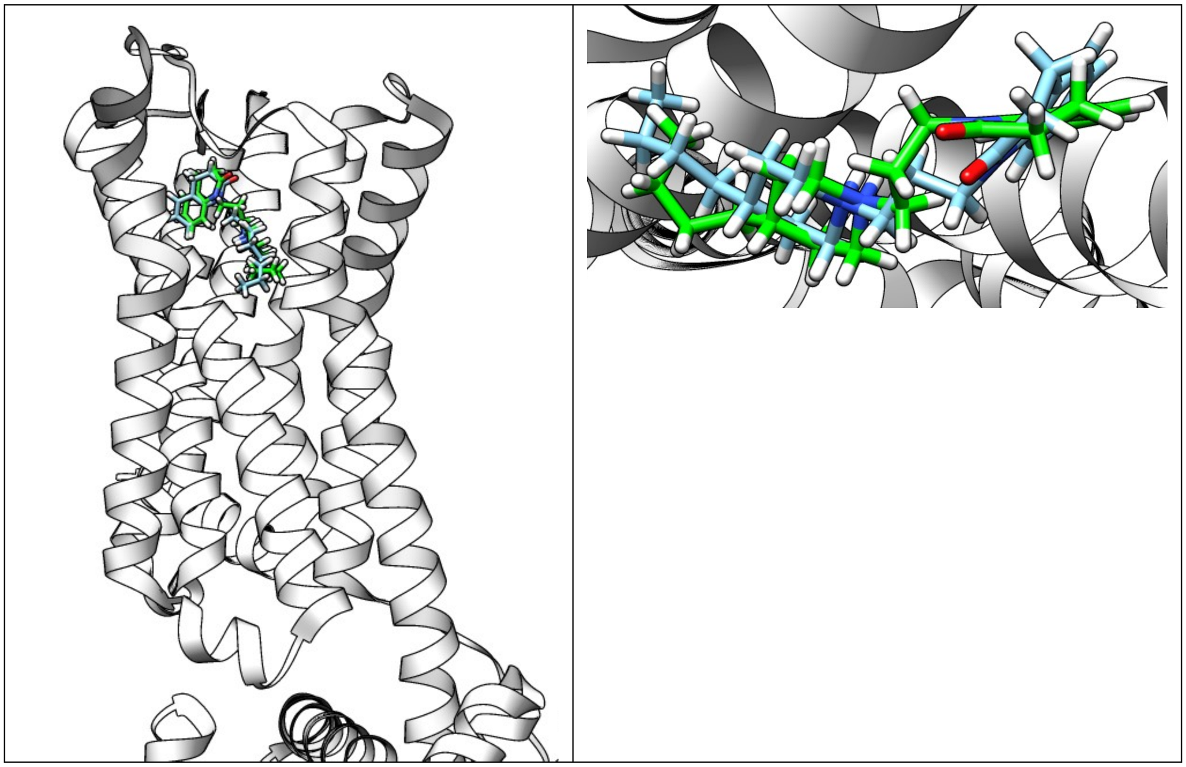

Figure-S4 Crystal structure of the M1R- 77-LH-28-1 complex (PDB:6ZFZ) with the overlapping ligand pose obtained from docking (RMSD=0.85 Å). The conformation obtained from the docking calculation is green, and the crystal conformation is cyan.

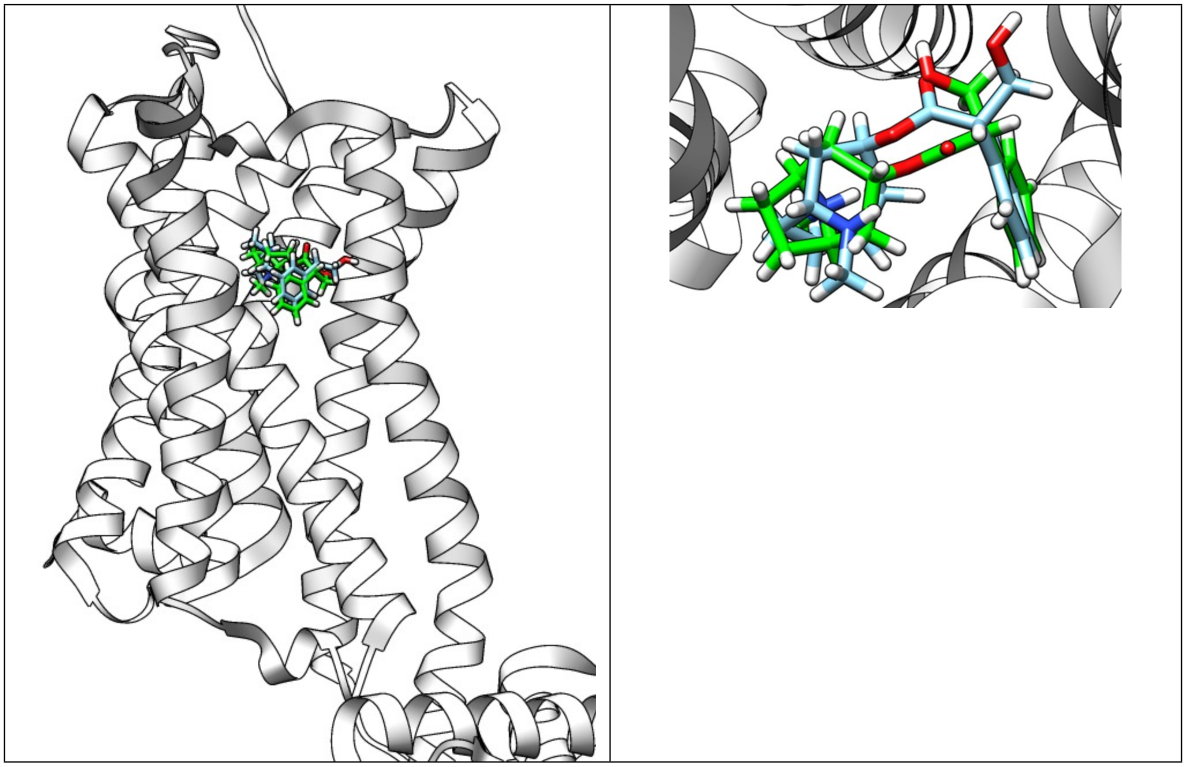

Figure-S5 Crystal structure of the M1R-atropine complex (PDB:6WJC) with the overlapping ligand pose obtained from docking (RMSD=1.2 Å). The conformation obtained from the docking calculation is green, and the crystal conformation is cyan.

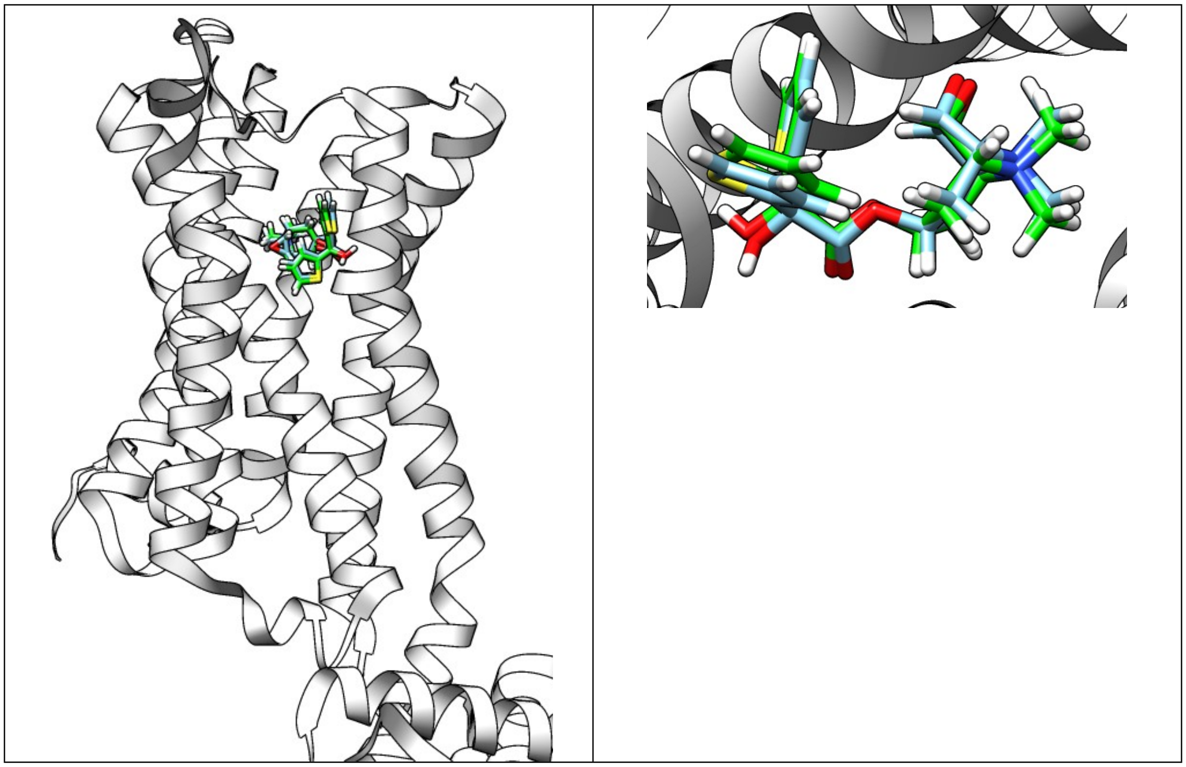

Figure-S6 Crystal structure of the M1R-tiotropium complex (PDB:5CXV)) with the overlapping ligand pose obtained from docking (RMSD=0.29 Å). The conformation obtained from the docking calculation is green, and the crystal conformation is cyan.

Figure-S7 Molecular descriptors obtained from SwissADME *and* ChemDes.

Descriptor Family	Molecular descriptors	Web server
Physicochemical	molecular weight, num. heavy atoms, fraction csp3, num. rotatable bonds, num. h-bond acceptors, num. h-bond donors, molar refractivity	SwissADME
Lipophilicity	log po/w (ilogp), log po/w (xlogp3), log po/w (wlogp), log po/w (mlogp), log po/w (silicos-it), consensus log po/w	SwissADME
Water Solubility	log s (esol), log s (ali), log s (silicos-it), solubility, class	SwissADME
Pharmacokinetics	gi absorption, bbb permeant, p-gp substrate, cyp1a2 inhibitor, cyp2c19 inhibitor, cyp2c9 inhibitor, cyp2d6 inhibitor, cyp3a4 inhibitor, log kp (skin permeation)	SwissADME
Druglikeness	lipinski, ghose, veber, egan, muegge, bioavailability score	SwissADME
Medicinal Chemistry	pains, brenk, leadlikeness, synthetic accessibility	SwissADME
Constitutional	molecular weight (not including h), count of hydrogen atoms, count of halogen atoms, count of hetero atoms, count of heavy atoms, count of atoms, count of cl atoms, count of br atoms, count of i atoms, count of c atoms, count of p atoms, count of s atoms, count of o atoms, count of n atoms, number of rings, number of rotatable bonds, number of h-bond donors, number of h-bond acceptors, number of single bonds, number of double bonds, number of triple bonds, number of aromatic bonds, number of all atoms, average molecular weight(not including h), molecular path counts of length 1-6 (pc1-pc6)	ChemDes
Connectivity	valence molecular connectivity chi index for path order 0-10, 0xv, 1xv, 2xv, 3xpv, 4xpv, 5xpv, 6xpv, 7xpv, 8xpv, 9xpv, 10xpv, valence molecular connectivity chi index for three cluster, valence molecular connectivity chi index for four cluster, valence molecular connectivity chi index for path/cluster, valence molecular connectivity chi index for cycles of 3-6 (3xvch), 4xvch, 5xvch, 6xvch, simple molecular connectivity chi indices for path order 0-10, 0x, 1x, 2x, 3xp, 4xp, 5xp, 6xp, 7xp, 8xp, 9xp, 10xp, simple molecular connectivity chi indices for three cluster, simple molecular connectivity chi indices for four cluster, simple molecular connectivity chi indices for path/cluster, simple molecular connectivity chi indices for cycles of 3-6 (3xch), 4xch, 5xch, 6xch, mean chi1 (randic) connectivity index, the difference between chi3c and chi4pc, the difference between chi0v and chi0, the difference between chi1v and chi1, the difference between chi2v and chi2, the difference between chi3v and chi3, the difference between chi4v and chi4, the difference between chiv3c and chiv4pc	ChemDes
Basak	information content with order 0 proposed by basak, information content with order 1,2,3,4,5,6 proposed by basak, complementary information content with order 0 proposed by basak, structural information content with order 1, 2, 3, 4, 5, 6 proposed by basak, complementary information content with order 0, 1, 2, 3, 4, 5, 6 proposed by basak	ChemDes
Topology	weiner index, average wiener index, balaban, harary number, schiultz index, graph distance index, platt number, xu index, polarity number, pogliani index, ipc index, bertzct, gutman molecular topological index based on simple vertex degree, zagreb index with order 1-2, modified zagreb index with order 1-2, quadratic index, largest value in the distance matrix, radius based on topology, petitjean based on topology, the logarithm of the simple topological index by narumi, harmonic topological index proposed by narnumi, geometric topological index by narumi, arithmetic topological index by narumi, total information index on molecular size, total information index on atomic composition, total information index on distance equality, mean information index on distance equality, total information index on vertex equality, logarithm of the simple topological index by narumi, harmonic topological index proposed by narnumi, geometric topological index by narumi, gravitational topological index based on topological distance, gutman molecular topological index based on valence vertex degree(log10)	ChemDes
Kappa	kappa alpha index for 1 bonded fragment, kappa alpha index for 2 bonded fragment, kappa alpha index for 3 bonded fragment, kier molecular flexibility index, molecular shape kappa index for 1, 2 and 3 bonded fragment	ChemDes
Burden	burden descriptors based on atomic mass, burden descriptors based on atomic volumes, burden descriptors based on atomic electronegativity, burden descriptors based on polarizability	ChemDes
E-state	Sum of E-State of atom type S1, S2, S3, S4, S5, S6, S7, S8, S9, S10, S11, S12, S13, S14, S15, S16, S, 17, S18, S19, S20, S21, S22, S23, S24, S25, S26, S27, S28, S29, S30, S31, S32, S33, S34, S35, S36, S37, S38, S39, S40, S41, S42, S43, S44, S45, S46, S47, S48, S49, S50	ChemDes
Moran	moran autocorrelation descriptors based on atom mass, van der waals volume, sanderson electronegativity, polarizability	ChemDes
Geary	geary autocorrelation descriptors based on atom mass, van der waals volume, sanderson electronegativity, polarizability	ChemDes
Molecular property	molar refractivity, logp value based on the crippen method, square of logp value based on the crippen method, topological polarity surface area, unsaturation index, hydrophilic index	ChemDes
Moreau-Broto autocorrelation	moreau-broto autocorrelation descriptors based on atom mass, atsm1, atsm2, atsm3 atsm4, atsm5, atsm6, atsm7, atsm8, moreau-broto autocorrelation descriptors based on atomic van der waals volume, atsv1, atsv2, atsv3, atsv4, atsv5, atsv6, atsv7, atsv8, moreau-broto autocorrelation descriptors based on atomic sanderson electronegativity, atse1, atse2, atse3, atse4, atse5, atse6, atse7, atse8, moreau-broto autocorrelation descriptors based on atomic polarizability, atsp1, atsp2, atsp3, atsp4, atsp5, atsp6, atsp7, atsp8	ChemDes
Charge	most positive charge on H atom, C atom, N atom, O atom, most negative charge on H atom, C atom, N atom, O atom, most positive charge in a molecule, most negative charge in a molecule, sum of squares of charges on H atom, C atom, N atom, O atom, sum of squares of charges on all atoms, mean of positive charges, total of positive charges, mean of negative charges, total of negative charges, mean of absolute charges, total of absolute charges, relative positive charge, Relative negative charge, submolecular polarity parameter, local dipole index	ChemDes
MOE-type	topological polar surface area based on fragments, labute's approximate surface area, moe-type descriptors using slogp and mr contributions and surface area contributions, moe-type descriptors using partial charges and surface area contributions, moe-type descriptors using estate indices and surface area contributions, moe-type descriptors using surface area contributions and estate indices	ChemDes

Figure-S8 Data values of agonists of M1-AChR obtained from the QSAR model.

Molecule	Name	Volume [Å³]	Y Exp.	Y-Calc	Y-Pred	Err.Calc.	Err.Pred.
1	77-LH-28-1	379.5	8.7	7.52	7.31	-1.18	-1.39
2	NNC 11-1607	564.73	8.6	8.66	9.01	0.06	0.41
3	AZD6088	416.01	8.3	7.84	7.76	-0.46	-0.54
4	SPP1	454.12	7.67	8.13	8.24	0.46	0.57
5	AC-260584	375.46	7.39	7.48	7.49	0.09	0.1
6	xanomeline	299.97	7.3	6.63	6.54	-0.67	-0.76
7	sabcomeline	207.12	6.7	5.3	5.21	-1.4	-1.49
8	GSK-1034702	339.04	6.5	7.1	7.19	0.6	0.69
9	arecaidine propargyl ester	202.15	6.4	5.22	5.14	-1.18	-1.26
10	oxotremorine	235.81	5.75	5.74	5.74	-0.01	-0.01
11	arecoline	172.67	5.7	4.72	4.61	-0.98	-1.09
12	(-)-aceclidine	181.6	5.4	4.87	4.83	-0.53	-0.57
13	oxotremorine-M	229.41	5.35	5.65	5.66	0.3	0.31
14	cevimeline	204.46	5.3	5.25	5.25	-0.05	-0.05
15	pilocarpine	222.06	5.1	5.53	5.56	0.43	0.46
16	McN-A-343	291.67	5	6.52	6.71	1.52 *	1.71 *
17	milameline	178.32	4.8	4.82	4.82	0.02	0.02
18	acetylcholine	171.6	4.6	4.7	4.71	0.1	0.11
19	methylfurmethide	185.82	4.6	4.94	4.97	0.34	0.37
20	(-)-YM796	213.06	4.55	5.39	5.44	0.84	0.89
21	carbachol	163.41	4.25	4.56	4.6	0.31	0.35
22	furtrethonium	167.71	4.1	4.63	4.7	0.53	0.6
23	bethanechol	181.39	4	4.87	4.94	0.87	0.94

Figure-S9 Data values of antagonists of M1-AChR obtained from the QSAR model

Molecule	Name	Volume [Å³]	Y Exp.	Y-Calc	Y-Pred	Err.Calc.	Err.Pred.
1	QNB	358.4	10.7	8.75	8.66	-1.95	-2.04
2	tiotropium	395.12	10.34	8.94	8.87	-1.4	-1.47
3	N-methyl scopolamine	330.56	9.9	8.45	8.37	-1.45	-1.53
4	glycopyrrolate	349.19	9.85	8.67	8.61	-1.18	-1.24
5	umeclidinium	475.09	9.8	8.49	7.56	-1.31	-2.24
6	propantheline	407.36	9.7	8.94	8.89	-0.76	-0.81
7	ipratropium	365.56	9.55	8.81	8.78	-0.74	-0.77
8	Telenzepine	372.03	9.4	8.85	8.83	-0.55	-0.57
9	4-DAMP	362.79	9.3	8.79	8.77	-0.51	-0.53
10	Biperiden	354.08	9.3	8.72	8.69	-0.58	-0.61
11	atropine	309.15	9.1	8.13	8.06	-0.97	-1.04
12	Benzatropine	348.43	9	8.66	8.65	-0.34	-0.35
13	scopolamine	312.32	9	8.18	8.13	-0.82	-0.87
14	trihexyphenidyl	350.58	8.9	8.68	8.67	-0.22	-0.23
15	dicyclomine	363.83	8.61	8.8	8.81	0.19	0.2
16	oxybutynin	387.97	8.45	8.92	8.94	0.47	0.49
17	tolterodine	406.22	8.4	8.94	8.98	0.54	0.58
18	pirenzepine	360.26	8.15	8.77	8.8	0.62	0.65
19	amitriptyline	327.15	7.8	8.41	8.44	0.61	0.64
20	solifenacin	387.35	7.8	8.92	8.97	1.12	1.17
21	VU0255035	394.36	7.8	8.94	8.99	1.14	1.19
22	dosulepin	328.22	7.7	8.42	8.46	0.72	0.76
23	darifenacin	460.81	7.65	8.66	9.06	1.01	1.41
24	himbacine	393.32	6.9	8.93	9.03	2.03 *	2.13
25	(S)-dimetindene	340.13	6.7	8.57	8.67	1.87	1.97
26	AZD6088	416.01	8.3	8.93	8.99	0.63	0.69
27	Xanomeline	299.97	7.3	7.96	8.01	0.66	0.71
28	Oxotremorine-M	235.81	5.75	6.38	6.45	0.63	0.7
29	(-)-aceclidine	181.6	5.4	4.46	4.05	-0.94	-1.35
30	Pilocarpine	222.06	5.1	5.94	6.07	0.84	0.97
31	Milameline	178.32	4.8	4.33	4.1	-0.47	-0.7
32	(-)-YM796	213.06	4.55	5.64	5.84	1.09	1.29

Figure-S10. BIOVIA Discovery Studio Visualizer analysis. Comparative analysis of the interaction types and amino acids involved at M1AChR.

Figure-S10.1 The crystal structure of the M1 receptor with iperoxo bound (PDB code: 6OIJ) was used as a template to assess the different M1R-ligand interactions.

Molecule	Hydrophobic contact				Carbon Hydrogen Bond				Electrostatic		Distance (Å)
Iperoxo	Tyr404	Tyr106	Trp378	Tyr381	Tyr404	Ser109	Tyr106	Asp382	Asp105	Tyr404	Ser109 (2.9)
(-)-aceclidine	—	—	—	—	✓	✓	—	—	✓	✓	Ser109 (3)
(-)-YM796	Ala193, Ala196	Val113,Phe197	✓	—	Tyr106	—	✓	—	Tyr106	—	Tyr106 (2.5)
77-LH-28-1	Ala193, Val385,Trp157,Cys407	✓	✓	✓	—	—	—	✓	—	—	Asn382 (2.6),Trp157 (2.7)
AC-42	Ala193,Val385,Leu183,Trp157,Phe182,Cys407	✓	—	—	—	✓	✓	Gln110	—	—	Asn382 (2.9)
AC-260584	Ala193,Val385,Trp157,Cys407	✓	—	✓	—	✓	✓	✓	—	—	Ser109 (3), Trp157 (2.6)
acetylcholine	✓	✓	—	✓	Asp105	✓	—	—	✓, Tyr106	✓	Trp157 (2.9)
arecaidine propargyl ester	✓	✓	Cyst407	✓	—	✓	✓	Tyr381	—	—	Asn382 (3.2)
arecoline	—	✓	✓	—	—	Ala193	✓	—	—	—	Gln110 (2.3)
AZD6088	—	Tyr85	Trp400	—	Glu397	—	—	—	—	—	Gln177 (2.8)
bethanechol	✓	✓	—	✓	—	✓	—	—	✓	Tyr106,Tyr381	Gln110 (1.9),Trp157 (2.7)
carbachol	✓	✓	—	—	Asp105	✓	—	—	✓	✓, Tyr106	Gln110 (2.9)
cevimeline	Ala193,Ala196,Val385,Trp157	—	—	✓	Gln110	—	—	—	—	—	Gln110 (2.4)
furtrethonium	✓	✓	✓	—	—	✓	✓	Asp105	✓	✓, Tyr106	Ser109 (3.1)
GSK-1034702	Trp400,Tyr179,Val395	—	—	—	Gln177	—	—	—	Glu397	—	Tyr179 (2.7)
HTL9936	Tyr179, Leu86	—	—	—	Trp400	—	—	—	Glu397	Tyr179	Glu397 (2.9), Lys392 (1.9)
LY593093	Trp400,Tyr179,Glu397,Thr398,Leu86,Leu186	—	—	—	Glu397	—	—	—	Glu397	—	Tyr85 (2.9)
McN-A-343	✓	✓	Cyst407	✓	Thr189	✓	Tyr381	✓	—	—	Gln110 (2.9)
methylfurmethide	✓	✓	Cyst407,Trp157	✓	—	Gln110	✓	—	—	—	Gln110(3.2), Tyr106(3.4)
milameline	—	—	—	—	Gln110	Ala196	—	—	—	—	Gln110(3.4), Ala196(3.3)
NNC 11-1585	✓	—	—	—	Glu397	Ser388	—	—	Glu397	—	Gln177 (3.2)
NNC 11-1607	Trp400,Tyr179,Tyr85,Leu86	—	—	—	Glu397	—	—	—	Lys392	Trp400	Gly89 (1.8)
oxotremorine	✓	✓	—	—	✓	—	✓	—	—	—	Gln110 (1.9)
oxotremorine-M	✓	—	—	—	✓	✓	—	Gln110	✓	✓	Ser109 (2.9)
Pentylthio-TZTP	✓	—	Tyr85,Leu102	—	Glu397	—	—	—	Glu397	Trp400	Glu397 (2.9)
pilocarpine	✓	—	✓, Cyst407,Ala196	✓	Asn382	—	—	—	—	—	Tyr404 (2.9)
sabcomeline	✓	—	—	—	Asn382	—	—	—	✓	✓, Tyr106	Gln110 (2.9)
Xanomeline	Ala193,Ala196,Val385,Trp157,Phe182,Tyr381	✓	—	✓	Asn382	—	—	—	Asp105	Tyr106	Ser109 (2.7)

Figure-S10.1 Continue

Molecule	Pi Cation	Pi sigma	Pi-Alkyl				Conventional Hydrogen Bond		Pi-Pi Stacked	Pi-Pi T-shaped	Pi anion	Halogen (Fluorine)	Pi-Sulfur	Salt Bridge	Amide-Pi Stacked
Iperoxo	Tyr404	Tyr404	—	—	—	—	—	—	—	—	—	—	—	—	—
(-)-aceclidine	✓	—	—	—	—	—	—	—	—	—	—	—	—	—	—
(-)-YM796	Tyr106	—	Phe197	Trp378	—	—	—	—	—	—	—	—	—	—	—
77-LH-28-1	—	—	Trp157	Cys407	—	—	Trp157	—	Tyr106	Tyr381, Trp378	—	—	—	—	—
AC-42	—	Tyr106	Trp157	Cys407	Phe182	—	Asn382	—	Tyr106	—	—	—	—	—	—
AC-260584	—	—	Trp157	Cys407	—	—	Ser109	Trp157	Tyr106	Tyr381	—	—	—	—	—
acetylcholine	✓, Tyr106	✓, Tyr106,Tyr381	—	—	—	—	Trp157	—	—	—	—	—	—	—	—
arecaidine propargyl ester	—	Tyr106	Tyr106	Tyr381	Tyr404	—	Asn382	—	—	—	—	—	—	—	—
arecoline	—	—	—	—	—	—	Gln110	—	—	—	—	—	—	—	—
AZD6088	—	Tyr85	Trp400	—	—	—	Gln177	—	—	—	—	—	—	—	—
bethanechol	Tyr106, Tyr381	✓, Tyr106,Tyr381	—	—	—	—	Trp157	Gln110	—	—	—	—	—	—	—
carbachol	✓, Tyr106	✓, Tyr106	—	—	—	—	Gln110	—	—	—	—	—	—	—	—
cevimeline	—	—	Trp157	Tyr381	—	—	Gln110	—	—	—	—	—	—	—	—
furtrethonium	✓, Tyr106	✓	—	—	—	—	—	—	Tyr106	Trp378	—	—	—	—	—
GSK-1034702	—	Trp400	Trp400	—	—	—	Tyr179	—	—	Tyr179	—	Ser388	—	Glu397	—
HTL9936	Tyr179	Tyr179	—	—	—	—	Glu397	Lys392	—	—	—	—	—	—	—
LY593093	Lys392	Trp400	Leu86	Leu183	—	—	Tyr85	—	Tyr179	Trp400	Glu397	Leu183,Ser388	—	—	Glu397, Thr398
McN-A-343	—	—	Tyr404	Tyr381	Cys407	—	Gln110	—	Tyr106	—	—	—	—	—	—
methylfurmethide	—	✓, Trp157	Tyr381	Cys407	—	—	—	—	Tyr106	—	—	—	—	—	—
milameline	—	—	—	—	—	—	—	—	—	—	—	—	—	—	—
NNC 11-1585	—	—	—	—	—	—	Gln177	—	—	Tyr404	Glu397	—	—	—	—
NNC 11-1607	Lys392,Trp400	—	Leu86	—	—	—	Gly89	—	Tyr85	Trp400, Tyr179	—	—	—	—	—
oxotremorine	—	—	—	—	—	—	Gln110	Cys407	—	—	—	—	—	—	—
oxotremorine-M	✓	✓	—	—	—	—	—	—	—	—	—	—	—	—	—
Pentylthio-TZTP	Trp400	Trp400	Tyr404	Tyr85	—	—	Glu397	Tyr85	—	—	Glu397	—	Trp400	—	—
pilocarpine	—	Tyr404,Tyr381	Trp378	Tyr381	Ala196	—	Tyr408	—	—	—	—	—	—	—	—
sabcomeline	✓, Tyr106	✓	—	—	—	—	Gln110	—	—	—	—	—	—	—	—
Xanomeline	Tyr106	Tyr106	Trp157	Tyr381	Ala196	Phe182	Ser109	—	—	Trp157	—	—	—	—	—

Figure-S10.2 The crystal structure of the M1 receptor with 77-LH-28-1 bound (PDB code: 6ZFZJ) was used as a template to assess the different M1R-ligand interactions.

**Molecule**	**Hydrophobic contact**							**Carbon Hydrogen Bond**			**Electrostatic**		**Distance (Å)**
77-LH-28-1	Cys407	Tyr381	Trp378	Tyr106	Ser109	Tyr82	Tyr404	Asp105	—	—	Asp105	Tyr404	Asp105 (1.9)
(-)-aceclidine	—	—	—	✓	—	✓	✓	Ala101	—	—	✓	—	Ala101(2.6)
(-)-YM796	Tyr85	—	—	—	—	✓	—	—	—	—	✓	—	—
AC-42	—	✓	✓	✓	—	✓	—	—	—	—	✓	✓	—
AC-260584	Trp157	Ala101	✓	✓	—	—	✓	Cys178	—	—	—	✓	Asp105 (1.95)
acetylcholine	Tyr85	—	—	—	—	✓	—	Cys178	—	—	Tyr85	—	Cys178 (3.7)
arecaidine propargyl ester	—	—	—	✓	—	✓	✓	Ser78	Tyr408	—	✓	—	Ser78 (3.5),Tyr408(3.5)
arecoline	Unfavorable												
AZD6088	Trp157	Ala101,Leu102,Cys178,	—	—	—	—	—	—	—	—	✓	✓	Cys178 (2.4)
bethanechol	—	✓	✓	✓	—	—	✓	Tyr381	—	—	✓	Tyr106	Tyr381(3.5)
carbachol	Tyr85	—	—	—	—	✓	—	—	—	—	Tyr85	—	Ser78 (2.9),Tyr408(2.3)
cevimeline	Tyr85	—	—	—	—	✓	✓	—	—	—	✓	—	—
furtrethonium	Cys178	—	—	—	—	✓	—	Ser78	—	—	✓	—	Ser78 (3.8)
Iperoxo	—	—	—	✓	—	✓	✓	Ser78	—	—	✓	—	Ser78 (3.7)
LY593093	—	Trp400,Leu102	—	✓	—	—	✓	—	—	—	✓	—	Asp105 (2.3)
McN-A-343	—	Ala101,Leu81,Phe77	—	✓	—	✓	—	✓	Ser109	—	✓	—	Asp105 (3.3),Ser109(3.4)
methylfurmethide	Tyr85	Cys178	—	—	—	✓	✓	—	—	—	✓	—	—
milameline	—	—	—	—	—	✓	✓	✓	—	—	—	—	Asp105 (3.5)
NNC 11-1585	✓	—	—	✓	—	✓	✓	—	—	—	—	—	—
NNC 11-1607	Pro186	—	—	—	—	✓	✓	Tyr106	—	—	✓	—	Tyr106(3.4)
oxotremorine	—	—	—	✓	—	✓	✓	—	—	—	—	—	—
oxotremorine-M	—	—	—	✓	—	✓	✓	Tyr408	—	—	✓	—	Tyr408(3.7)
Pentylthio-TZTP	Tyr85	Cys178,Trp91,Leu102	—	✓	—	✓	✓	—	—	—	✓	Tyr106	Asp105 (2.2)
pilocarpine	Tyr85	Ala101,Trp91,Leu102	—	✓	—	✓	✓	Tyr408	—	—	—	—	Tyr408(3.3)
sabcomeline	—	—	—	—	—	✓	✓	Tyr106	—	—	—	—	Tyr106(3.6)
xanomeline	Tyr85	Cys178,Trp91	—	✓	—	✓	✓	—	—	—	✓	Tyr381	—
GSK-1034702	Tyr85	—	—	—	—	—	✓	Tyr179	—	—	✓	—	Tyr179(2.8)
HTL9936	—	✓	✓	✓	—	—	✓	✓	—	—	—	✓	Asp105 (2.4)

Figure-S10.2 Continue															
**Molecule**	**Pi Cation**	**Pi sigma**	**Pi-Alkyl**				**Salt bridge**	**Conventional Hydrogen Bond**			**Pi-Pi Stacked**	**Pi-Pi T-shaped**	**Pi anion**	**Halogen**	**Pi-Sulfur**
77-LH-28-1	Tyr404	—	Trp378	Tyr381	—	—	Asp105	—	—	—	—	—	—	—	—
(-)-aceclidine	—	—	—	—	—	—	—	Ala101	—	—	—	—	—	—	—
(-)-YM796	—	—	Tyr82	Tyr85	—	—	—	—	—	—	—	—	—	—	—
AC-42	✓	—	✓	✓	Tyr106	—	—	—	—	—	—	Tyr82	—	—	—
AC-260584	✓	Tyr404	✓	Ala101	Tyr106	Trp157	✓	—	—	—	—	—	—	—	—
acetylcholine	Tyr85	Tyr85	—	—	—	—	—	—	—	—	—	—	—	—	—
arecaidine propargyl ester	—	—	Tyr404	—	—	—	—	—	—	—	—	—	—	—	—
arecoline															
AZD6088	✓	—	Trp157	—	—	—	✓	Cys178	—	—	—	—	—	—	—
bethanechol	Tyr106	Tyr106	—	—	—	—	—	Tyr381	Thr196	Asp382	—	—	—	—	—
carbachol	Tyr85	Tyr85	—	—	—	—	—	Ser78	Tyr408	—	—	—	—	—	—
cevimeline	—	—	Tyr82	Tyr85	Tyr404	—	—	—	—	—	—	—	—	—	—
furtrethonium	—	—	Cys178	—	—	—	—	—	—	—	—	—	—	—	—
Iperoxo	—	—	—	—	—	—	—	—	—	—	—	—	—	—	—
LY593093	—	—	—	—	—	—	—	Asp105	Ser78	—	Tyr404	Tyr106,Trp400	Asp105	—	Cys178,Cys407
McN-A-343	Tyr106	Tyr106	Phe77	—	—	—	—	—	—	—	—	—	—	—	—
methylfurmethide	—	—	Tyr82	Tyr85	Tyr404	Cys178	—	—	—	—	Tyr82	—	—	—	—
milameline	—	Tyr404	—	—	—	—	—	—	—	—	—	—	—	—	—
NNC 11-1585	—	—	Cys407	—	—	—	—	—	—	—	—	—	—	—	—
NNC 11-1607	—	—	Pro186	—	—	—	—	Ala101	Thr189	—	Tyr82	Tyr404	—	—	Tyr404
oxotremorine	—	—	—	—	—	—	—	Tyr404	—	—	—	—	—	—	—
oxotremorine-M	—	—	—	—	—	—	—	Tyr404	—	—	—	—	—	—	—
Pentylthio-TZTP	Tyr106	Tyr106,Tyr85	Tyr82	Trp91	Leu102	—	✓	—	—	—	Tyr404	—	—	—	Tyr106,Tyr408
pilocarpine	—	—	Tyr82,Tyr85	Trp91	Tyr106,Tyr404	Ala101,Leu102	—	Tyr106	—	—	—	—	—	—	—
sabcomeline	—	—	—	—	—	—	—	—	—	—	—	—	—	—	—
xanomeline	Tyr381	Tyr85	Tyr82	Trp91	—	—	—	—	—	—	Tyr404	Tyr106			Tyr106
GSK-1034702	—	—	Tyr85	—	—	—	—	—	—	—	—	Tyr404	—	—	—
HTL9936	✓	Tyr106,Tyr404	✓	✓	—	—	✓	—	—	—	—	—	—	—	—

Figure-S10.3 The crystal structure of the M1 receptor with atropine bound (PDB code: 6WJC) was used as a template to assess the different M1R-ligand interactions.

**Molecule**	**Hydrophobic contact**						**Carbon Hydrogen Bond**			**Electrostatic**		**Distance (Å)**
atropine	Val113	Ala196	Tyr381	Tyr106	Ser109	Tyr404	Asp105	Thr192	—	Asp105	Trp378	Asn382 (2.4), Ser109 (3.1)
(S)-dimetindene	—	✓	Trp378,Cys407	—	—	✓	Ala196	—	—	—	—	Thr192 (2.2)
4-DAMP	Val385	✓	✓	✓	Ala193	✓	✓	Tyr404	—	✓	Tyr404	Asn382 (2.4)
aclidinium	Trp378	Leu183	✓	✓	—	—	—	—	—	—	—	Tyr381(2.3)
AFDX384	Trp400	✓	—	✓	—	✓	Tyr404	Tyr106	—	Glu401	—	Asn382 (2.4), Cys 407 (2.7)
amitriptyline	Trp378	✓	✓	—	Ala193	—	—	—	—	—	—	Asp105(2.2)
AQ-RA 741	✓	✓	✓	✓	Trp378	✓	Ala193	Asp382	—	—	—	Ala193(2.6),Asn382(2.9)
Benzatropine	—	✓	✓	✓	Ala193	—	✓	—	—	✓	✓	Ser109 (2.6)
Biperiden	Val385	✓	✓	✓	Ala193	✓	—	—	—	Trp378	Tyr381	—
darifenacin	✓	✓	—	✓	—	✓	—	—	—	—	—	Asn382 (1.8), Thr192 (1.9)
dicyclomine	✓	✓	Trp378	✓	Ala193	✓	✓	Ser109	—	✓	Tyr404	Asn382 (2.5)
dosulepin	Trp378	✓	—	—	Ala193	✓	—	—	—	✓	Tyr404	—
droxidopa	—	✓	✓	✓	—	—	—	—	—	✓	Asn382, Thr192	Asn382 (2.5), Asp105 (2.6), Thr192 (2.8)
glycopyrrolate	✓	✓	—	✓	Trp378	✓	✓	Tyr404	Ser109	✓	Tyr404	Asn382 (2.4)
himbacine	Trp378	—	✓	✓	Cys407	✓	—	—	—	—	—	—
ipratropium	—	✓	—	✓	Tyr408	✓	—	—	—	✓	—	—
N-methyl scopolamine	—	✓	—	✓	—	✓	—	—	Tyr404	✓	Tyr404	Tyr404(3.2)
oxybutynin	—	✓	✓	✓	Ala193	✓	✓	—	—	—	—	Asn382 (2.2), Ser109 (2.6)
pirenzepine	—	✓	✓	✓	Ala193,Val385,Tyr408	✓	—	Ser109	—	✓	Tyr404,Tyr408	Asn382 (2)
propantheline	—	✓	✓	✓	Ala193,Tyr408	✓	—	—	—	✓	Tyr404	Asn382 (1.9)
QNB	Trp378	✓	✓	✓	Ala193	—	—	—	—	✓	—	Asn382 (2.5)
revefenacin	—	✓	—	✓	—	—	Thr189	✓	Cys178	—	—	Asn382 (2.8), Thr192(2.1), Glu401 (2.2)
scopolamine	✓	✓	✓	✓	—	—	—	✓	—	✓	✓	Asn382 (2.3), Tyr408 (2.7)
solifenacin	—	✓	—	✓	Ala193	—	—	Asn382	—	—	Tyr404	Asn382 (2.6)
Telenzepine	✓	✓	✓	✓	Ala193,Trp378,Tyr408	—	—	Ser109	—	✓	Tyr408	Asn382 (1.9)
tiotropium	—	✓	✓	✓	Trp157,Ala193	✓	Ala193	Tyr404	—	✓	Tyr404,Tyr381	Asn382 (2.4)
tolterodine	—	✓	—	✓	Cys407,Trp378,Tyr408	✓	Asn382	—	—	—	—	Asn382 (3.4)
trihexyphenidyl	✓	✓	✓	✓	Ala193,Trp378	✓	—	—	—	—	✓	—
umeclidinium	✓	✓	—	✓	Cys407,Ile180	—	—	—	—	—	—	Asn382(3)
VU0255035	—	—	✓	✓	—	✓	Tyr381	✓	Glu401	—	—	Tyr106(2.4),Thr192(2.7)

Figure-S10.3 Continue

Molecule	Pi Cation	Pi sigma	Pi-Alkyl			Conventional Hydrogen Bond			Pi-Pi Stacked	Pi-Pi T-shaped	Pi anion	Pi-Sulfur	Pi lone pair
atropine	Trp378	—	Val113	Ala196	—	Asn382	Ser109	—	—	—	—	—	—
(S)-dimetindene	—	Trp378	Cys407	✓	—	Thr192	—	—	—	Trp378,Tyr404	—	—	—
4-DAMP	Tyr404	Tyr106, Tyr404	Val385	✓	Ala193	✓	—	—	—	Tyr381	—	—	—
aclidinium	—	—	Leu183	—	—	Tyr381	—	—	—	Trp378,Tyr381	—	Cys407	—
AFDX384	—	—	Trp400	✓	Tyr404	✓	Cys407	—	—	Tyr106	—	Cys407	—
amitriptyline	—	—	—	✓	Ala193	—	—	—	—	Trp378,Tyr381	—	—	—
AQ-RA 741	—	—	✓	✓	—	—	—	—	—	Trp378	—	Cys407	—
Benzatropine	✓	—	—	✓	Ala193	✓	—	—	—	Tyr381	—	—	—
Biperiden	✓, Ty381	Tyr404	Val385	Ala193	—	—	—	—	—	Tyr381	—	—	—
darifenacin	—	—	✓	✓	Tyr404	✓	Thr192	—	Tyr106	—	—	—	—
dicyclomine	Tyr404	Tyr106, Tyr404	Trp378	—	—	✓	—	—	—	—	—	—	—
dosulepin	Tyr404	Tyr404	Ala193	✓	—	—	—	—	—	Trp378	—	Trp378	—
droxidopa	—	—	—	—	—	✓	Thr192	Asp105	Tyr106	—	—	—	—
glycopyrrolate	Tyr404	Trp378	✓	✓	Tyr106	✓	—	—	—	Trp378	—	—	—
himbacine	—	—	Trp378	Tyr404	Tyr381	—	—	—	—	—	—	—	—
ipratropium	—	Tyr404,Tyr408	—	✓	—	—	—	—	—	—	—	—	—
N-methyl scopolamine	Tyr404	Tyr404	—	✓	—	—	—	—	—	—	—	—	—
oxybutynin	—	Tyr404	—	Ala193	—	✓	✓	—	—	Tyr381	—	—	—
pirenzepine	Tyr404,Tyr408	Tyr404,Tyr408	Val385	✓	Ala193	✓	—	—	—	Tyr381	—	—	—
propantheline	Tyr404	Tyr404,Tyr408,Tyr106	—	✓	Ala193	✓	—	—	—	Tyr381	—	—	—
QNB	Tyr404	Trp378	—	✓	Ala193	✓	—	—	—	Trp378,Tyr381	—	—	—
revefenacin	—	—	—	✓	—	✓	Thr192	Glu401	Tyr106	Tyr106	—	—	Tyr106
scopolamine	✓	—	✓	✓	—	✓	Tyr408	—	—	—	—	—	—
solifenacin	Tyr404	—	Ala193	✓	—	✓	—	—	—	—	—	—	—
Telenzepine	Tyr408	Trp378,Tyr408	✓	✓	Ala193	✓	—	—	—	Trp378,Tyr381	—	Trp378	—
tiotropium	Tyr404,Tyr381	Tyr404	—	✓	Ala193	✓	—	—	Trp157	Tyr381	—	Trp378,Tyr381	—
tolterodine	—	—	Cys407	✓	Trp378,Tyr408	Tyr106	—	—	—	Tyr106,Ty404	—	—	—
trihexyphenidyl	✓	Tyr404	✓	✓	Tyr381	—	—	—	—	Trp378	—	—	—
umeclidinium	—	—	✓	✓	Cys407,Ile180	Asn382	—	—	—	—	—	—	—
VU0255035	—	—	—	—	Tyr404	Tyr106	Thr192	—	Tyr404	Tyr381	—	Tyr381	—

Figure-S10.4 The crystal structure of the M1 receptor with tiotropium bound (PDB code: 5CXV) was used as a template to assess the different M1R-ligand interactions.

**Molecule**	**Hydrophobic contact**						**Carbon Hydrogen Bond**			**Electrostatic**			**Distance (Å)**	
tiotropium	Tyr404	Tyr106	Tyr381	Ala196	Ala193	Other	Tyr404	—	—	Tyr404	Asp105	Other	Asn382 (2)	—
(S)-dimetindene	✓	✓	—	—	✓	Cys407	—	—	—	—	—	—	—	—
4-DAMP	—	✓	—	✓	✓	Trp378,Val113	—	—	Ser109	✓	✓	Tyr106	Asn382 (2)	—
aclidinium	✓	✓	✓	—	—	Ile180,Leu183,Val385,Cys407	—	Asp382	—	—	—	—	Asn382(3.7)	—
AFDX384	✓	—	✓	✓	—	Ty82,Trp101	—	Tyr106	—	—	—	—	Asn382 (2)	Tyr381 (2.8), Thr189 (2.7)
amitriptyline	✓	✓	—	✓	—	Trp378,Val113,Cys407	—	—	—	—	—	—	—	Thr189 (2.5)
AQ-RA 741	—	✓	—	✓	✓	Trp400,Ile180	—	Tyr106	—	—	—	Tyr106	—	Tyr106(2.9)
atropine	✓	✓	—	✓	—	—	—	Asp105	—	✓	✓	Tyr106	—	Asp105(3.2)
Benzatropine	—	—	—	✓	✓	Trp378,Val113	—	—	—	—	✓	Trp378	—	—
Biperiden	✓	—	✓	✓	✓	—	—	—	—	—	—	Trp378	—	—
darifenacin	—	—	—	—	—	Trp400,Leu86	Cys178	—	—	✓	—	Glu401	—	Trp400 (2.2)
dicyclomine	—	✓	—	✓	✓	Trp378	—	—	—	—	✓	Trp378	—	Tyr106(2.9)
dosulepin	—	✓	—	✓	—	Trp378,Tyr381	—	—	—	—	—	—	—	Thr189 (2.3)
droxidopa	—	—	—	✓	—	—	—	—	—	—	—	—	Thr189(2.2)	Thr192 (2.5), Tyr106 (3)
glycopyrrolate	—	✓	—	✓	—	Trp378,Val113	✓	Asp105	Ser109	✓	✓	—	Asn382 (2.2)	—
himbacine	—	—	—	—	—	Trp400	Tyr85	—	Cys178	—	—	—	Tyr85(3.7)	Cys178(3.5)
ipratropium	—	—	—	✓	—	—	—	—	—	—	—	Tyr106	Thr192(2.3)	—
N-methyl scopolamine	✓	—	✓	✓	—	—	✓	—	Ala193	✓	—	Tyr381	Tyr404(3.6)	Ala193(3)
oxybutynin	✓	✓	✓	✓	—	Cys407	—	—	Ala193	—	—	—	Asn382 (3)	Tyr106(2.2)
pirenzepine	✓	—	—	—	—	—	Ser184	Glu401	Leu183	—	—	Trp400	Glu401(2.2)	Leu183(3.6),Ser184(3.5)
propantheline	✓	✓	✓	✓	✓	—	—	Asp382	—	—	—	—	Asn382 (1.9)	—
QNB	—	✓	—	✓	—	Trp378,Val113	—	—	—	—	✓	—	Asn382 (2.3)	—
revefenacin	—	—	—	—	—	Trp400,Tyr404,Leu86,Leu183	—	—	Pro186	—	—	Glu401	Cys178(2.6)	—
scopolamine	✓	—	—	✓	—	Trp378	✓	—	—	✓	—	Tyr381	Thr192(2.7)	—
solifenacin	✓	✓	—	✓	✓	Trp378,Trp157	—	—	—	—	✓	—	Asn382 (2.5)	Ser109(2-5)
Telenzepine	✓	—	—	—	—	Trp400	—	—	Glu401	—	—	Glu401	Ile180(3)	—
tolterodine	✓	—	—	✓	—	Trp378	—	—	—	—	—	—	—	—
trihexyphenidyl	—	—	—	✓	✓	Trp378	—	—	—	✓	✓	Tyr106	—	—
umeclidinium	—	✓	—	✓	—	Thr189,Trp378,Val113,Pro186	Tyr106	—	—	—	—	—	Tyr381 (2.2)	—
VU0255035	✓	✓	—	✓	✓	—	—	—	—	—	—	—	Tyr381 (2.2)	—

Figure-S10.4 Continue

Molecule	Pi Cation	Pi sigma		Pi-Alkyl			Conventional Hydrogen Bond			Pi-Pi Stacked	Pi-Pi T-shaped		Sulfur-X	Pi-Sulfur			Pi anion
tiotropium	Tyr404	Tyr381	Tyr404	Ala196	Ala193	—	Asn382	—	—	—	—	—	Thr192	Trp378	Tyr381	Phe197	—
(S)-dimetindene	—	—	—	—	✓	Cys407	—	—	—	Tyr404,Tyr106	—	—	—	—	—	—	—
4-DAMP	✓, Tyr106	—	Tyr106	✓	✓	Val113	✓	—	—	—	Trp378	—	—	—	—	—	—
aclidinium	—	—	—	Cys407,Val385	Ile180	Leu183	✓	—	—	Tyr404,Tyr106	Tyr381	—	—	—	—	—	—
AFDX384	—	—	—	✓	Tyr404,Tyr82	Trp101	✓	Tyr381,Thr189	Tyr381	Tyr381	—	—	—	Cys407	—	—	—
amitriptyline	—	—	Trp378	✓	Trp378,Cys407,Val113	Ala193	—	Thr189	—	Tyr404,Tyr106	Trp378	Tyr381	—	—	—	—	—
AQ-RA 741	Tyr106	—	Tyr106	—	Ile180	—	—	—	—	Trp400	—	—	—	—	—	—	—
atropine	✓, Tyr106	—	✓	✓	—	—	—	—	—	—	—	—	—	—	—	—	—
Benzatropine	Trp378	—	—	✓	✓	Val113	—	—	—	—	Trp378	—	—	—	—	—	—
Biperiden	Trp378	—	✓	—	✓	—	—	—	—	—	Tyr381	—	—	—	—	—	—
darifenacin	✓	—	—	—	Trp400,Leu86	—	—	Trp400	—	—	—	—	—	—	—	—	Glu401
dicyclomine	Trp378	—	Tyr106	—	—	Trp378	—	Tyr106	—	—	—	—	—	—	—	—	—
dosulepin	—	—	—	✓	—	Trp378	—	Thr189	—	Tyr381	Tyr106	Trp378	—	Cys407	—	—	—
droxidopa	—	—	—	✓	—	—	—	Thr189,Thr192	Tyr106	—	—	Trp378	—	—	—	—	—
glycopyrrolate	✓	—	—	✓	Val113,Tyr106	—	✓	—	—	—	—	Trp378	—	—	—	—	—
himbacine	—	—	—	—	—	Trp400	—	—	—	—	—	—	—	—	—	—	—
ipratropium	Tyr106	—	—	✓	—	—	—	Thr192	—	—	—	—	—	—	—	—	—
N-methyl scopolamine	✓, Tyr381	✓	✓	✓	—	—	—	—	—	—	—	—	—	—	—	—	—
oxybutynin	—	—	—	✓	Tyr106,Tyr381,Tyr404	—	✓	—	Tyr106	—	—	—	—	—	—	—	—
pirenzepine	Trp400	—	—	—	—	—	—	—	—	Tyr404	—	—	—	—	—	—	—
propantheline	—	✓	✓, Tyr106	✓	✓	—	✓	—	—	—	—	—	—	—	—	—	—
QNB	✓	—	—	✓	—	Val113	✓	—	—	—	Tyr106	Trp378	—	—	—	—	—
revefenacin	—	—	—	—	Leu86,Leu186	—	—	Pro186,Cys178	—	—	—	Trp400	—	—	—	—	Glu401
scopolamine	✓	✓	✓	✓	—	—	Tyr404	Thr192	—	—	Trp378	—	—	—	—	—	—
solifenacin	—	—	✓	✓	✓	—	✓	—	Ser109	Tyr106,Trp157	—	Trp378	—	—	—	—	—
Telenzepine	—	—	—	—	—	—	—	—	Ile180	Tyr404	Trp400	—	—	Trp400	—	—	—
tolterodine	—	—	—	✓	—	—	—	—	—	—	Tyr404	Trp378	—	Cys407	—	—	—
trihexyphenidyl	✓, Tyr106		—	✓	—	—	—	—	—	—	Trp378	—	—	—	—	—	—
umeclidinium	—		Thr189,Trp378	✓	—	Val113,Pro186	—	—	Tyr381	Trp378	✓	Trp378	—	Cys407	—	—	—
VU0255035	—		—	✓	—	Tyr106	—	—	Tyr381	Tyr404	—	—	—	—	—	—	—

Figure-S11. Scatterplots of predicted pKi values against experimental pKi values, and Williams plots for agonists of M1-AChR.

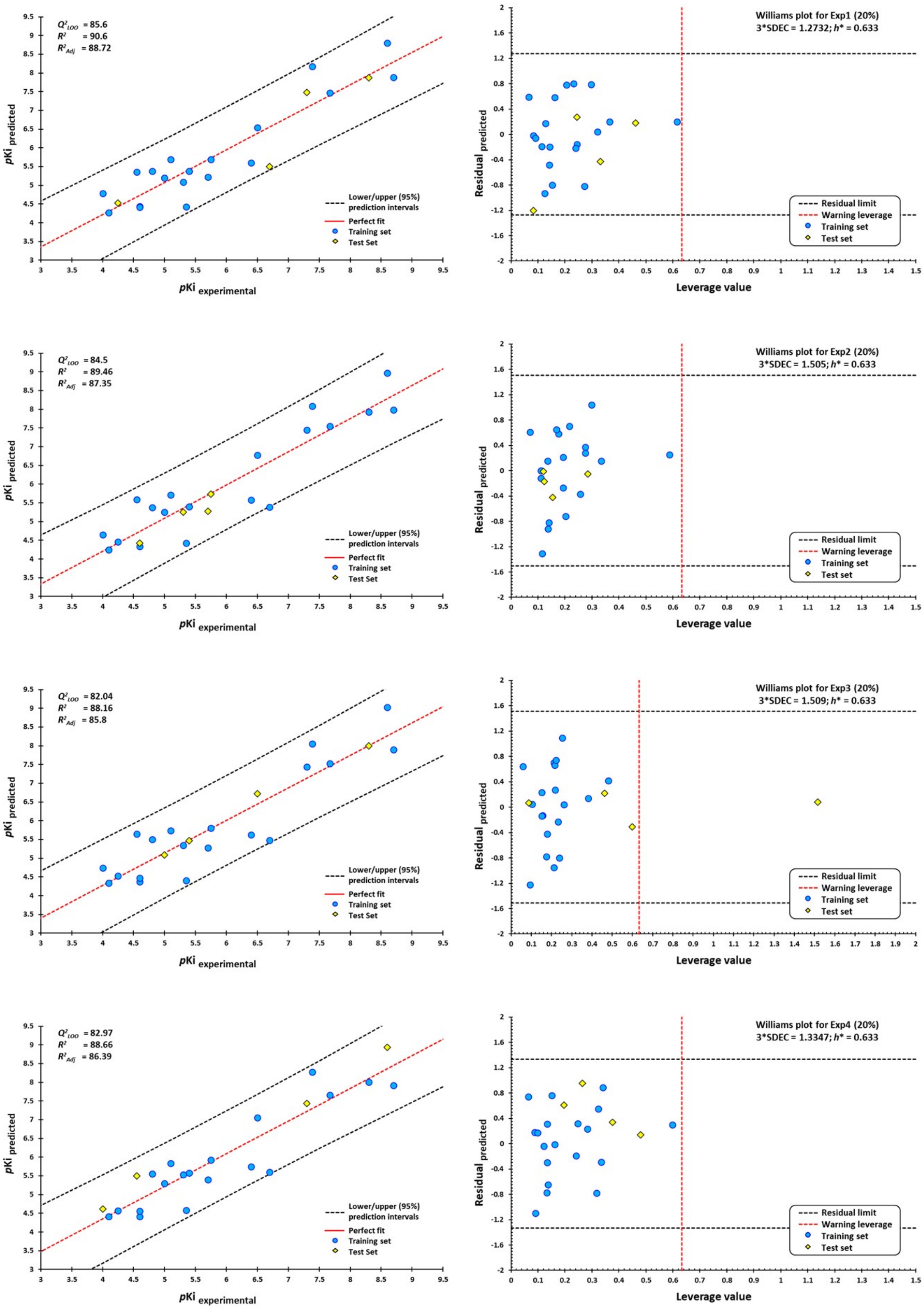

Figure-S12. Scatterplots of predicted pKi values against experimental pKi values, and Williams plots for antagonists of M1-AChR.

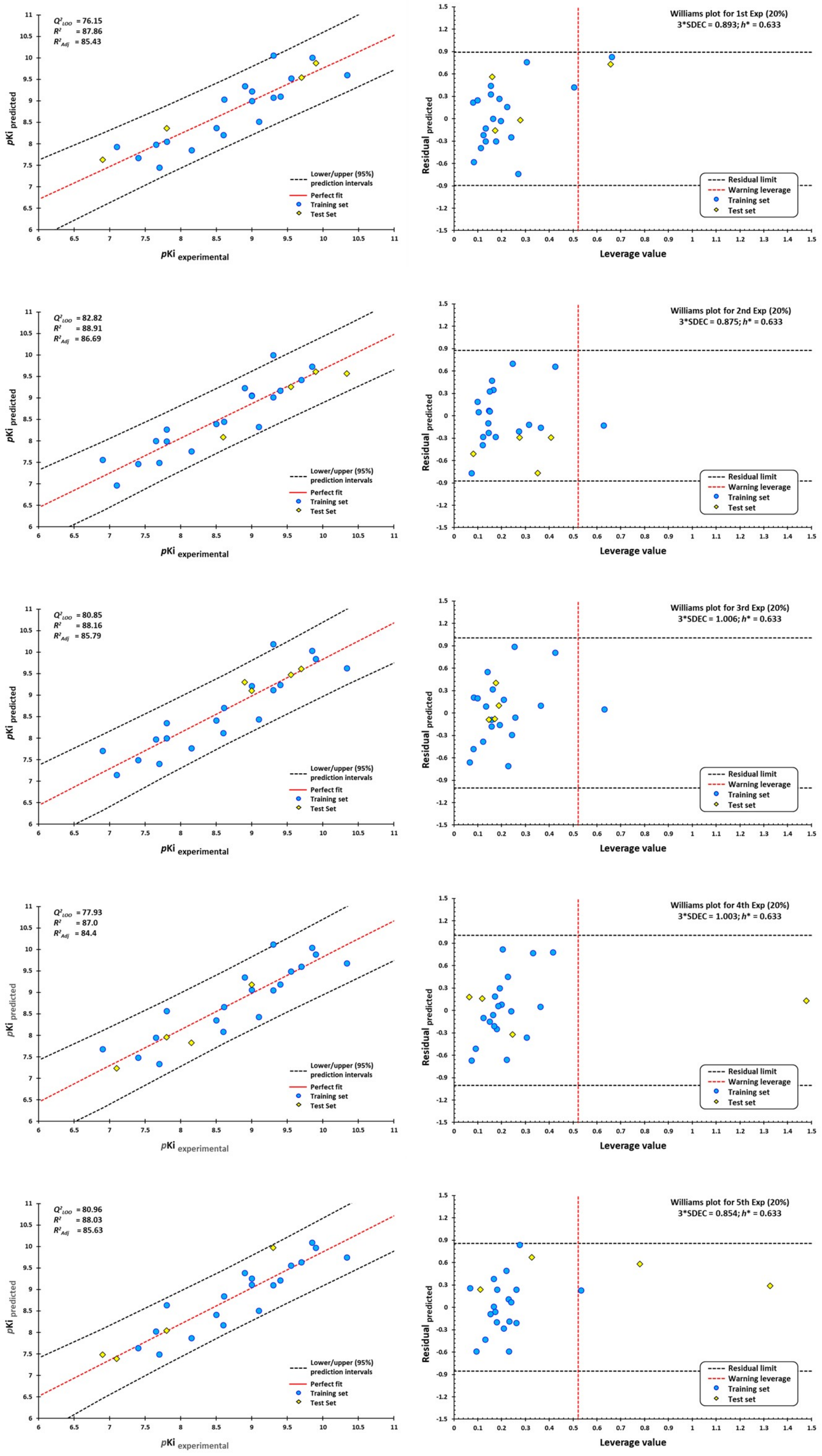

Supplement: RA-014-D3RA07380G-s001 [file RA-014-D3RA07380G-s001.pdf]
